# An Optimal Cost Effectiveness Study on Zimbabwe Cholera Seasonal Data from 2008–2011

**DOI:** 10.1371/journal.pone.0081231

**Published:** 2013-12-03

**Authors:** Tridip Sardar, Soumalya Mukhopadhyay, Amiya Ranjan Bhowmick, Joydev Chattopadhyay

**Affiliations:** 1 Agricultural and Ecological Research Unit, Indian Statistical Institute, Kolkata, West Bengal, India; 2 Department of Statistics, Visva Bharati University, Santiniketan, West Bengal, India; Northeastern University, United States of America

## Abstract

Incidence of cholera outbreak is a serious issue in underdeveloped and developing countries. In Zimbabwe, after the massive outbreak in 2008–09, cholera cases and deaths are reported every year from some provinces. Substantial number of reported cholera cases in some provinces during and after the epidemic in 2008–09 indicates a plausible presence of seasonality in cholera incidence in those regions. We formulate a compartmental mathematical model with periodic slow-fast transmission rate to study such recurrent occurrences and fitted the model to cumulative cholera cases and deaths for different provinces of Zimbabwe from the beginning of cholera outbreak in 2008–09 to June 2011. Daily and weekly reported cholera incidence data were collected from Zimbabwe epidemiological bulletin, Zimbabwe Daily cholera updates and Office for the Coordination of Humanitarian Affairs Zimbabwe (OCHA, Zimbabwe). For each province, the basic reproduction number (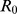) in periodic environment is estimated. To the best of our knowledge, this is probably a pioneering attempt to estimate 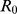 in periodic environment using real-life data set of cholera epidemic for Zimbabwe. Our estimates of 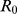 agree with the previous estimate for some provinces but differ significantly for Bulawayo, Mashonaland West, Manicaland, Matabeleland South and Matabeleland North. Seasonal trend in cholera incidence is observed in Harare, Mashonaland West, Mashonaland East, Manicaland and Matabeleland South. Our result suggests that, slow transmission is a dominating factor for cholera transmission in most of these provinces. Our model projects 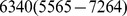 cholera cases and 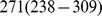 cholera deaths during the end of the epidemic in 2008–09 to January 1, 2012. We also determine an optimal cost-effective control strategy among the four government undertaken interventions namely promoting hand-hygiene & clean water distribution, vaccination, treatment and sanitation for each province.

## Introduction

Cholera is still a burning problem in underdeveloped and developing countries causing morbidity and mortality. In Zimbabwe, one of the most severe cholera outbreaks occurred in 2008–2009, that had been attributed as the worst African outbreaks in terms of its high case fatality rate (CFR) and short-time extensive spread in some provinces. The outbreak, beginning in Chitungwiza, had duration from August 2008 to July 2009, ultimately ended with 98,592 reported cases and 4,288 reported deaths [1]. These massive outbreaks happened mainly due to Zimbabwe's poor health care system, shortage of good-quality food and clean drinking water [2]. An economic crisis within this period accelerated the deterioration of the country's infrastructure, including a breakdown of basic municipal services (such as sewage treatment and water supply in many areas) and medical facilities [3].

The provinces of Zimbabwe experienced a total of 2101 cholera cases over the period, 17th October, 2009 to 30th June, 2011 [4], [5]. The substantial number of cholera cases in some provinces, *e.g.* Manicaland, Mashonaland West, Masvingo, Midlands, etc., both during and after the epidemic in 2008–09, indicate a plausible presence of seasonal forcing in cholera incidence in some of the provinces.

Well strategic deployment of cholera intervention/interventions in Zimbabwe may reduce future cases and deaths, although the projected effect of available cholera interventions is debatable [6]. A lot of suggestions have come out for preventing the cholera outbreak in those regions. Many regional and international organizations suggest providing clean water, hand-hygiene (Soap) promotion and construction & promotion of sanitary systems. Other groups are arguing for the vaccination program, although some experts suggest that the effect of vaccination will be modest [7]. Several professionals have also recommended usage of rehydration therapy for mild infections (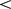
10% bodyweight loss) and usage of antibiotics (Erythromycin, Doxycycline and Ringer Lactate) for severe cases (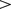
10% bodyweight loss) to reduce morbidity [8], cost of productive time loss due to illness, and bacterial shedding [9]. With proper treatment of cholera cases, the CFR should remain below 1% [10]. However, in terms of cost effectiveness, cholera vaccination is by far most costly intervention[11] with US$1,658 to US$8,274 yields one DALY (Disability-adjusted life year) and gaining that same year through promoting hand-hygiene need US$3.35, making hand-hygiene the cheapest among cholera interventions [12]. Even though, promoting hand-hygiene heavily depends upon the availability of clean water. So an optimal balance among different types of interventions may significantly reduce the number of cholera cases and deaths at a minimal cost. Thus, a well-coordinated effort and an effective response to control an outbreak are the most important tasks.

To control future epidemics, a good understanding of cholera transmission dynamics is crucial and mathematical models can be utilized as a potential tool [6], [13]–[15]. Some earlier studies on cholera are based on the assumption of constant transmission rate between human and bacterial population over time [6], [13]–[15] but in food or waterborne infections, the role played by temporal forcing is more subtle and interesting. There is strong evidence that the multi annual dynamics of cholera are interlinked with long-term environmental factors [16]–[18].

To capture the presence of possible seasonal pattern within the data of reported cholera cases, we include the periodicity factor in our model. With these backdrops, we modified the model proposed by Hartley *et al.* [15] to include periodic slow-fast transmission and fitted to Zimbabwe's weekly cholera seasonal data starting from 2008–2009 epidemics to June 2011. Daily and weekly data were collected from Zimbabwe epidemiological bulletin [4], Zimbabwe Daily cholera updates [5] and Office for the Coordination of Humanitarian Affairs Zimbabwe [19]. The basic reproduction number (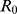) carries information about the persistence of a disease [20], [21]. It is inversely proportional to the mean age of (first) infection; greater it is shorter the generation time, and the disease transmission will be more explosive [21], [22]. Aforesaid data was used to estimate 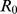 in periodic environment, for all provinces across the country. To the best of our knowledge, this is probably the pioneering attempt to estimate 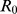 in periodic environment using the real data set of cholera epidemic. We perform a statistical test suggested by Roger[23], using weekly cholera incidence data from each province to justify the presence of seasonal trend. We also study the existence of any underlying pattern of temporal forcing in slow-fast transmission rate with the seasonality in cholera incidence, as observed in some provinces. We provide forecasts of cumulative cases and deaths from the end of epidemic in 2008–09 to 1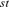 Jan 2012 for different provinces in Zimbabwe and study the optimal intervention strategy/strategies by minimizing the cost of different cholera interventions.

## Materials and Methods

### Basic model structure

We modify the existing model [15] assuming temporal variations in two types of transmission rates (slow and fast). The existing model [15] assumes constant human population size (birth and death rates are equal), neglects the cholera-related death rate and assumes life time natural immunity to cholera if recovered. We have modified these assumptions in our model by incorporating variable human population size, cholera-related death rate and the effect of natural immunity loss to cholera (as it is now proven fact that natural immunity to cholera varies from less than one year to two years [24]). Our basic model is a system of five differential equations (see Equation 1 & 2) describing how individuals can move between different states of susceptibility or infection with cholera.

We categorize the total human populations at time 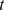 (denoted by 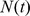), into susceptible 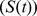, infected 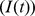, and recovered 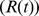 classes. A constant recruitment rate (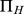) to human population, which is the product of human birth rate (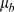) and initial entire human population size 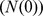, is considered. Individuals die naturally at a rate 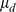. All newly recruited individuals are assumed to be susceptible.

A Recent study showed that freshly shed *V. cholerae* from human intestines are short-lived and hyper-infectious in nature [25]. It out competes other *V. cholerae* grown in vitro, by as much as 700-fold for at least the first 5 to 18 hours in the environment [25]. After the hyper-infectious stage, *V. cholerae* organisms lose their competitive advantage and become low-infectious. This hyper-infectivity is a key factor to understand the explosive nature of human-to-human transmission in cholera outbreaks. Based on this fact, we classify the bacterial populations in two states, one hyper-infectious 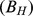 state for fast transmission and this hyper-infectious bacteria decay to become low-infectious 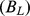 state after some time, which causes slow environmental transmission.

Susceptible individuals gain infection by consuming water contaminated with the low-infectious bacteria 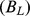 and the high-infectious bacteria 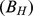 at rates 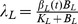 and 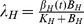, respectively. The subscripts 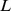 and 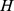 denote low infectious and high infectious cholera transmission. Here, 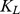 and 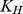 are half saturation constants of low-infectious and high-infectious bacterium respectively [15]. 
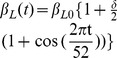
 and 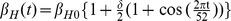 are the rates of ingesting low-infectious and high-infectious *V. cholerae* bacterium from the contaminated water, which are assumed to be time periodic with period 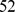 weeks. 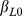, 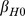 denote the minimum transmission rate of low and high infectious *V. cholerae* respectively from the contaminated water, and 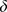 denotes the amplitude of seasonality. Infected individuals either have a natural death (at a rate 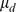) or die due to extreme loss of fluid from their body during infection (at a rate 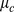) or recover naturally from cholera infection (at a rate 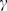). Recovered individuals are immune to reinfection, but this immunity wanes over time and eventually returns to the susceptible stage (at a rate 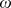). During the period of infection; infected individuals excrete *V. cholerae* into water reservoirs around them (at a rate 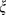). Since this bacterium is coming directly from infected human intestines, it is in hyper-infectious stage and decays, ultimately leads to low infectious stage (at a rate 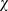). The natural death rate of low infectious bacterium is 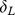.

**Figure 1:**
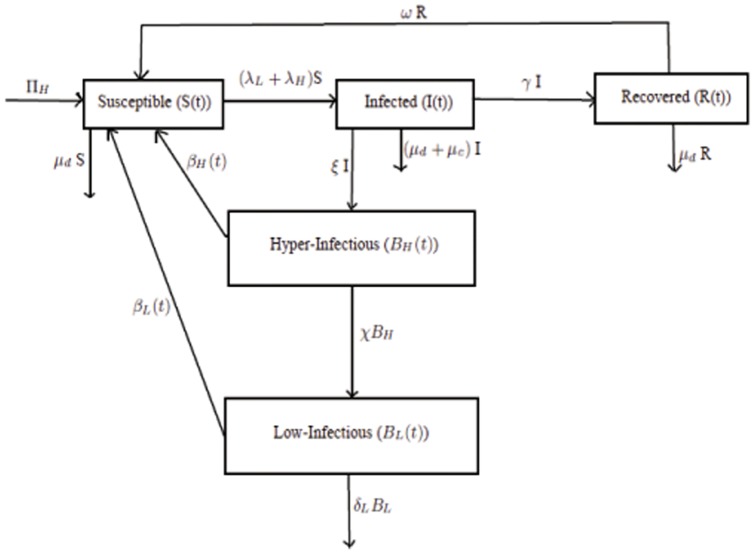
Cholera transmission model without any interventions.

Based on the above-mentioned assumptions, we construct the following system of non-linear differential equations:
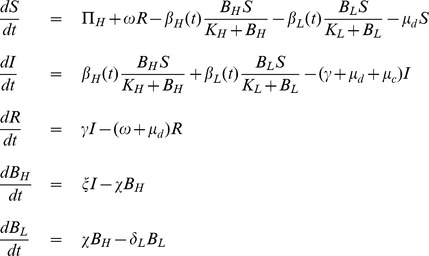
(1)where,



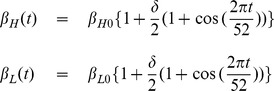
(2)Model parameters and their interpretations with some parameters' base values, taken from previous studies, are given in **Table S1**. A flow diagram of the basic cholera model (1) is also given in **Figure 1**.

### Source of data

Daily and weekly cholera incidence data for each province of Zimbabwe have been collected from Zimbabwe epidemiological bulletin [4], Zimbabwe Daily cholera updates [5] and Office for the Coordination of Humanitarian Affairs Zimbabwe [19] starting from 2008–2009 epidemics to June 2011. The data at the beginning of the epidemic are quite noisy. To smoothen out the initial fluctuations, the data is converted to weekly reported cases by aggregating daily case reports for the entire duration of the epidemic. Table 1 contains information about the starting points and the end points of the data for each province. Total numbers of data points vary across the provinces.

**Table 1:** Data Summary.

Province	Start date	End date	Number of data points	References
Harare	August 18, 2008	September 12, 2010	48	First 12 points from [19], next 30 points are from [5] and remaining 6 points are from [4]
Bulawayo	November 14, 2008	April 25, 2009	17	First 6 points from [19] and remaining 11 points are from [5]
Mashonaland West	September 21, 2008	March 27, 2011	61	First 11 points from [19], next 29 points are from [5] and remaining 21 points are from [4]
Mashonaland Central	November 14, 2008	May 30, 2010	34	First 5 points from [19], next 24 points are from [5] and remaining 5 points are from [4]
Mashonaland East	October 6, 2008	March 13, 2011	36	First 8 points from [19], next 25 points are from [5] and remaining 3 points are from [4]
Midlands	November 11, 2008	January 23, 2011	39	First 8 points from [19], next 24 points are from [5] and remaining 7 points are from [4]
Masvingo	November 13, 2008	June 26, 2011	55	First 5 points from [19], next 33 points are from [5] and remaining 17 points are from [4]
Manicaland	November 1, 2008	June 12, 2011	85	First 9 points from [19], next 34 points are from [5] and remaining 42 points are from [4]
Matabeleland South	November 13, 2008	April 4, 2010	23	First 8 points from [19], next 12 points are from [5] and remaining 3 points are from [4]
Matabeleland North	December 25, 2008	June 20, 2009	13	[5]

### Model calibration

To calibrate the basic model (1), we have considered the weekly reported cholera cases and deaths from each province of Zimbabwe starting from 2008–2009 epidemics to June 2011. The model is fitted to the cumulative number of cases and deaths obtained from the weekly counts in each province.

The key parameters estimated from the data are the average transmission rate of hyper-infectious Bacterium (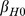), the average transmission rate of low-infectious Bacterium (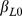), the amplitude of seasonality (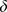), the mortality rate of human due to cholera infection (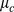) and the excretion rate of cholera infected individual (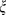). It is not realistic to assume the entire population of a province to be susceptible to cholera, as outbreaks generally occur in that part of the province where the basic amenities like proper drainage system, clean water and food are lacking. So, we first estimate the initial number of susceptible, infected and recovered human populations from the data by bounding the initial total human population size (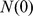) by the total population size of the province. The initial concentrations of hyper-infectious (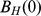) and low-infectious (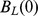) bacterium are estimated from the data. Our work also involves estimation of initial reported cases (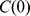) and deaths (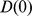) from the data since in some provinces the exact reported cases and deaths from the beginning of the epidemic were unknown due to reporting delays.

The cumulative cases and cumulative deaths from the cholera model (1) are given by:
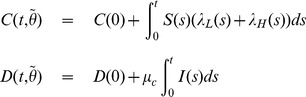
(3)


where 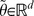 contains all the unknown variables of the model (1). We have 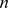 observations (cumulative cases and cumulative deaths) from our data at 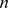 different weeks 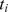 as 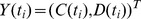, where 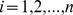 and 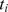 is the 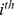 week in our data.

We assume independent Gaussian prior specifications for 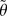:
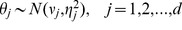
(4)


Let 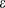 be the error when fitting cumulative quantities 
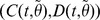
 from the model (1) to the observed data. Then 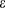 follows independent Gaussian distribution having unknown variance 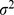 *i.e.* 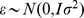. For the error variance a Gamma distribution is used as a prior for its inverse:
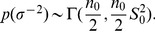
(5)where the prior parameters 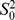 and 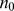 in (5) can be interpreted as the prior mean for 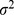 and the prior accuracy as imaginary observations.

We construct the sum of squares function as:
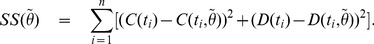
(6)


Posterior distribution of the model unknown variable 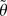 is generated using Delayed Rejection Adaptive Metropolis algorithm (DRAM) [26], [27] with an initial burn of 100000 iterations. MCMC toolbox in MATLAB written by Marko Laine [28] was used to estimate the unknown variable 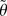 for the model (1). Geweke's Z-scores [29] were examined to ensure the chain convergence.

The advantage of using the cumulative over the weekly number of new cases in model calibration is that the former smoothes out known reporting delays on weekends and national holidays [14], [30].

### Seasonality

To justify whether the existence of any kind of seasonal forcing influence the number of cholera incidence in Zimbabwe provinces, a suitable statistical testing procedure is very much needed. The weekly data from 18th August, 2008 to 30th June, 2011 is taken into account for this purpose. We follow the test procedure suggested by Roger[23].

The entire span of almost 3 years is divided into 52 classes, corresponding to the 52 weeks of an year (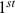 week starting from 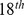 August, 2008) and the total number of cholera cases in week 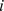 (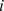 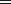 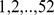) is denoted by 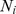. The probability that any one event belongs to 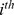 class is 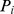, where
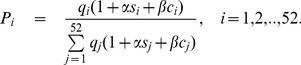
(7)where, 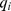 denotes the frequency for class 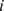 under the null hypothesis, 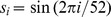, 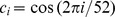, 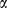 and 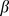 are the parameters of the model (7). 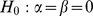 indicates the absence of seasonality and 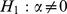 or 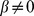 indicate the seasonality in cholera incidence.

The test statistics for testing 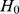 is of the form
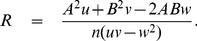
(8)


Where,
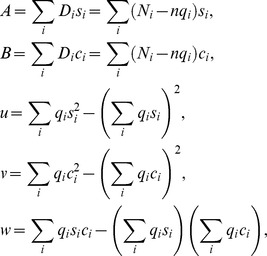



and 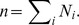


The test statistic 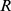 is asymptotically distributed as chi-square with 2 degrees of freedom.

### Estimating reproductive numbers in periodic environments

The Model (1) has a unique disease free equilibrium given by:
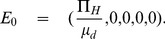
(9)


Following [31], we calculate the matrix of new infection from our system (1) as:
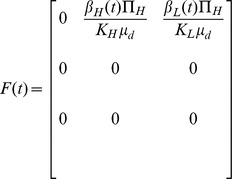
and the transmission matrix as:



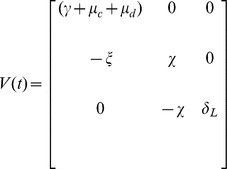



Let, 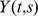, 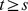 be the evolution operator of the linear 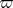-periodic system
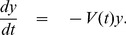
(10)


That is, for each 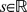, the 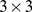 matrix 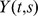 satisfies
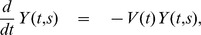
for all 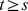 and 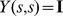, where 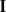 is the 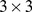 identity matrix.

Let 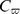 be the ordered Banach space of all 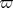-periodic functions from 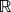 to 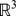 which is equipped with maximum norm 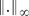 and the positive cone 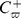 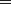 {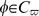: 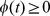, for all t in 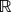}. Consider the following linear operator 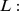 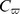 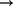 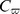 by
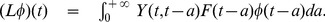
(11)


Following Wang and Zhao (2008) [31], we call 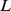 the next infection operator, and define the basic reproduction number (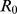) as:
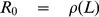
(12)where 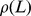 is the spectral radius of the operator 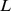 defined in Equation (11).

Motivated by the concept of the partial reproduction numbers defined by Mukandavire *et.al.* [14], we similarly define two partial reproduction numbers 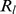 and 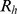 in periodic environment. The subscripts 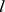 and 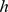 correspond to low infectious and high infectious transmission, respectively.

Basic reproduction number (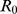) is the sum of two partial reproductive numbers- the one is arising from the contact between the human and low-infectious bacteria, which we denote as 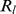 and the other one arising from the contact between the human and hyper-infectious bacteria, denoted as 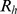. Using **Lemma 1** given in **Appendix S1** and the estimated parameter values (**Table S3 and Table S4**), we numerically estimate 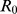, 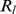 and 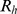 for each province. Details of the derivation procedure of 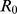, 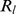 and 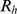 are given in **Appendix S1**. For uncertainty, we draw 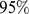 confidence interval around the estimated values. The following procedure is applied to derive the 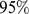 confidence intervals for 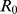, 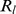 and 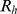, respectively.

We draw a sample of size 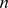 (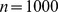) from the posterior distribution of 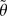 (set of model variables, which are estimated) using simple random sampling without replacement (SRSWOR) scheme. The posterior distribution of 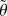 is depicted in **Figure S1 and Figure S2**. For each of the sample values of 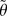, we estimate numerically the value of 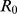 (using **Lemma 1**, **Appendix S1**). Thus, a vector of size 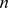, is generated for 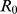. Curtailing the lower 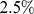 and the upper 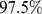 observations from the ordered vector of 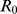, we obtain the 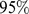 confidence interval for 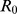. Applying the similar procedure we draw 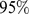 confidence intervals for two partial reproductive numbers, 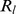 and 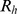, respectively.

### Projection of future cases and deaths

We project the number of cholera cases and deaths from the end of epidemic in 2008–09 to January 1, 2012. For uncertainty, we derive 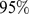 credible intervals around the estimates of future projected cases and deaths. To predict the number of cases and deaths for a particular province, we simulate the cholera model (1), using the known & estimated parameters (**Table S1 and Table S3**) and demographic parameters (**Table S4**), up to the end of epidemic in 2008–09, in that region. We obtain different demographic variables of human & pathogen (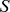, 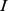, 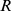, 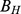 and 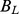), new cases and deaths corresponding to the end of epidemic in 2008–09. Using this information from the previous simulation as initial conditions and parameter values from **Table S1 and Table S3**, we simulate the model (1) to obtain predicted cases and deaths from the end of the epidemic in 2008–09 to January 1, 2012.

We used the following procedure to derive the 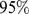 confidence intervals for projected cases and deaths. For each of the sample value of 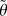 (see, section-**Estimating reproductive numbers in periodic environments**), we predict the number of cases and deaths using the above procedure. Thus, two vectors, each of size 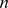, are generated for predicted cases and deaths, respectively. Curtailing the lower 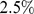 and the upper 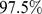 observations from the ordered vector of predicted cases and deaths we obtain the 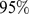 confidence intervals for projected cases and deaths, respectively.

### Model with different cholera interventions

Effect of four different types of cholera interventions namely hand-hygiene promotion & clean water supply, treatment using oral rehydration therapy & antibiotics, vaccination and sanitation are studied. We assume that hand-hygiene (soap) & clean water will reduce bacterial ingestion by a fraction 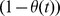, where 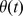 is the relative rate of reduction in bacterial ingestion per week using hand-hygiene & clean water supply. Vaccinated population is increased by a proportion 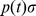 of the susceptible individuals, who are successfully vaccinated, where 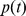 is the per week vaccination rate and 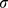 is the vaccine efficiency. Vaccinated population is decreased due to the waning of vaccine based immunity (at a rate 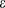) to become susceptible again and die (natural deaths) at a rate 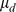. We assume that a proportion 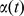 of the infected individuals receive treatment by oral rehydration salt (for 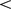 10% body weight loss) and by antibiotic (for 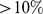 body weight loss) per week. Natural recovery rate of the treated person increases by relative rate of recovery 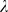. Since, excretion can be affected by the use of antibiotics [32], [33], the relative rate of shedding is reduced by a fraction 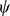 among the proportion of the infected individuals who receive antibiotic at a rate 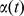 per week. Now proper sanitary and drainage system will prevent the human waste to contaminate the nearby water reservoirs. Invariably, sanitation will reduce excretion rate of human that contaminate the nearby reservoirs by a fraction 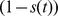, where 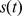 is the rate of reduction in human shedding per week by construction and promotion of sanitation. In Zimbabwe, 80% of the total populations have access to improved water source and 40% of the total populations have access to proper sanitation facilities [1]. Therefore, we assume maximum percentage reduction in bacterial ingestion rate through hand hygiene & clean water supply and reduction in human shedding possible by promoting sanitation in a week to be 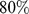 and 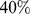, respectively. We also assume that maximum 70% of the infected individuals receive proper treatment in a week and maximum vaccination coverage possible in a week is about 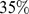 of the total susceptible population [34]. Effects of hand-hygiene & clean water, vaccination, treatment, sanitation and their different combinations are projected from the end of epidemic in 2008–09 to January 1, 2012.

System of non-linear differential equations representing the effect of different interventions on our basic model (1) is given as follows:
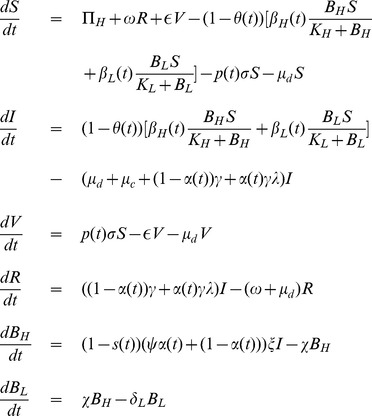
(13)


**Figure 2:**
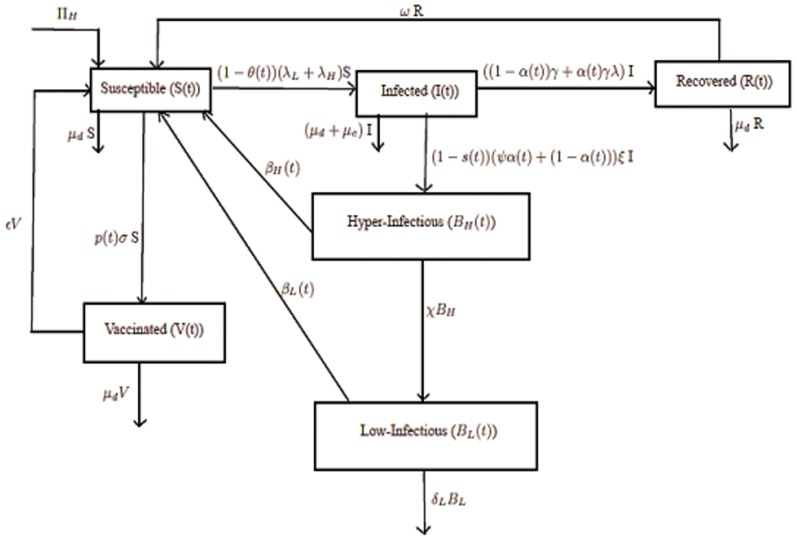
Cholera transmission model with different interventions.

Intervention parameters and their interpretations with some parameters' base values taken from earlier studies are given in **Table S2**. A flow diagram of the intervention model is depicted in **Figure 2**.

### An optimal intervention strategy

To determine the optimal intervention strategy/strategies (which reduce the number of cases and deaths projected from the end of the epidemic in 2008–09 to January 1, 2012, at a minimal cost), we define the following cost function:
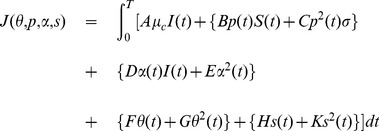
(14)where interventions are applied for 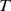 weeks. First term in the right-hand side of (14) represents the cost of cholera-related deaths and remaining terms are costs associated with the implementation of different interventions. Nonlinear terms in the objective function 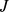 represent the costs of interventions in emergency situations. 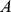, 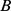, 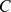, 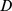, 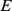, 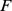, 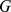, 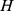 and 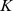 are fixed cost coefficients, given in the Table 2.

**Table 2:** Fixed cost-coefficients.

Notations	Interpretations	Year	Value (US$)	Reference
A	Cost of productive time lost per premature death (Calculated with life expectancy 73 years)	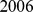	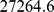	[56]
B	Cost of oral cholera vaccine (OCV) per fully immunized person	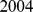	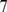	[57]
C	Cost of OCV per fully immunized person in high emergencies	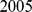	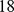	[57]
D	Cost of medicines and health centre consultation per mild/moderate case	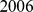	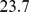	[56]
E	Cost of medicines and hospital admission per severe cholera cases	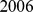	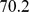	[56]
F	Cost of per percent reduction in bacterial ingestion rate by promoting hand-hygiene and water supply	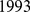	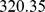	[12]
G	Cost of per percent reduction in bacterial ingestion rate by promoting hand-hygiene and water supply in high emergencies	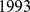	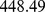	assumed 40% increase in normal cost
H	Cost of per percent reduction in human shedding by promoting sanitation (construction and promotion of latrine and drainage system)	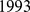	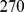	[12]
K	Cost of per percent reduction in human shedding by promoting sanitation (construction and promotion of latrine and drainage system) in high emergencies	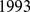	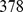	assumed 40% increase in normal cost

Fixed costs are transformed to subsequent intervention year costs by multiplying with a constant 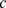, where 
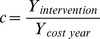
. 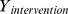 and 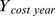 are the annual consumer price indices (US) when interventions are applied (*i.e.* from the end of epidemic in 2008–09 to January 1, 2012) and the annual consumer price index (US) of the years (see Table 2) respectively. 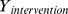 and 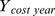 are in the same base 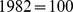, data of annual consumer price index (US) were collected from U.S. Bureau of Labor Statistics [35].

Our goal is to minimize the objective function 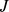 with respect to different control parameters 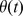, 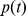, 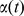 and 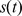 to determine an optimal intervention combination. This is a dynamic control problem and is solved directly by using the Pontryagin's Maximum Principle [36] and the method of steepest decent [37]. Minimization procedure of the objective function 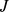 is briefly described in **Appendix S1**.

The average coverage percentages (per week) of hand-hygiene (soap) & clean water distributions, treatment and sanitation are estimated using the following formulas:
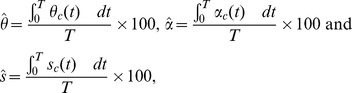



where, 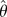, 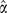 and 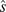, denote the average coverage percentages (per week) of hand-hygiene (soap) & clean water distributions, treatment and sanitation, respectively. 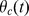, 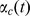 and 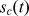 are the optimal rates of hand-hygiene (soap) & clean water distributions, treatment and sanitation, respectively, for which the cost function 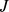 (see equation (14)) is minimum. 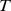 denotes the total number of weeks during which an intervention is applied.

Total coverage percentage of vaccination is estimated using the following formula:

Total vaccination coverage 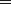 
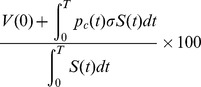
,

where, 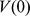 is the initial number of vaccinated individuals and 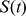 is the number of susceptible individuals at week 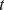. 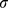 is the vaccine efficiency (**Table S2**). 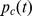 is optimal vaccination rate which minimizes the cost function 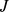 (see equation (14).

Cost per averted case for an intervention is calculated using the following formula:


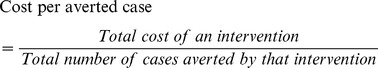


A 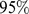 confidence interval for each of the following quantities are obtained following the same technique as explained in sections **Projection of future cases and deaths** and **Estimating reproductive numbers in periodic environments**: (1) cases that occurred in spite of applying an intervention, (2) total cost of an intervention and (3) cost per averted case.

## Results

**Figure 3:**
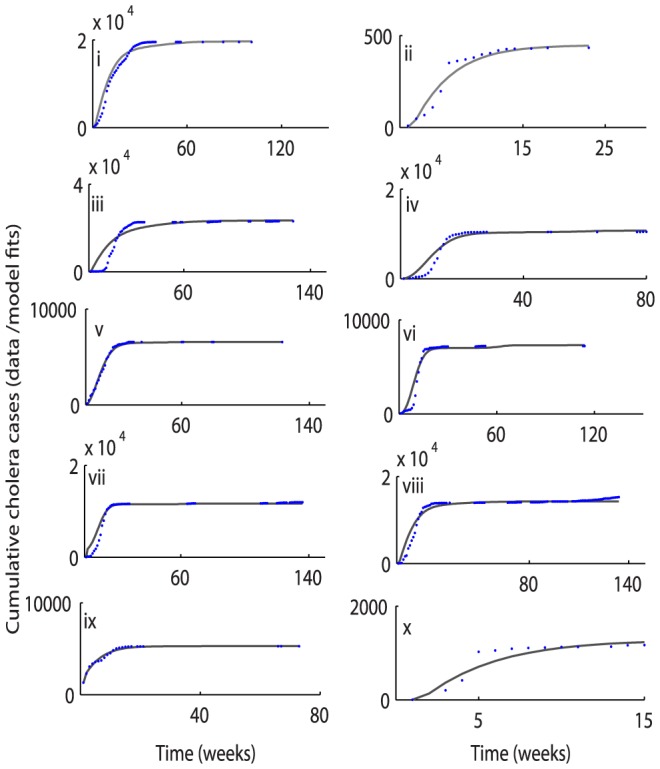
Province-wise cumulative cholera cases in Zimbabwe. The observed data points (available at some discrete time points over a time period, which varies across the study regions) are shown by blue circles while the solid lines depict the model solutions. The cumulative cholera cases from the model are plotted for each day of the time period (from the start to end week for the observed cholera data) using parameter values and initial conditions from **Table S3 and Table S4**. The above plots of cholera cases from the different provinces of Zimbabwe are as follows: (i) Harare; (ii) Bulawayo; (iii) Mashonland West; (iv) Mashonland Central; (v) Mashonland East; (vi) Midlands; (vii) Masvingo; (viii) Manicaland; (ix) Matabalend South; and (x) Matabalend North.

**Figure 4:**
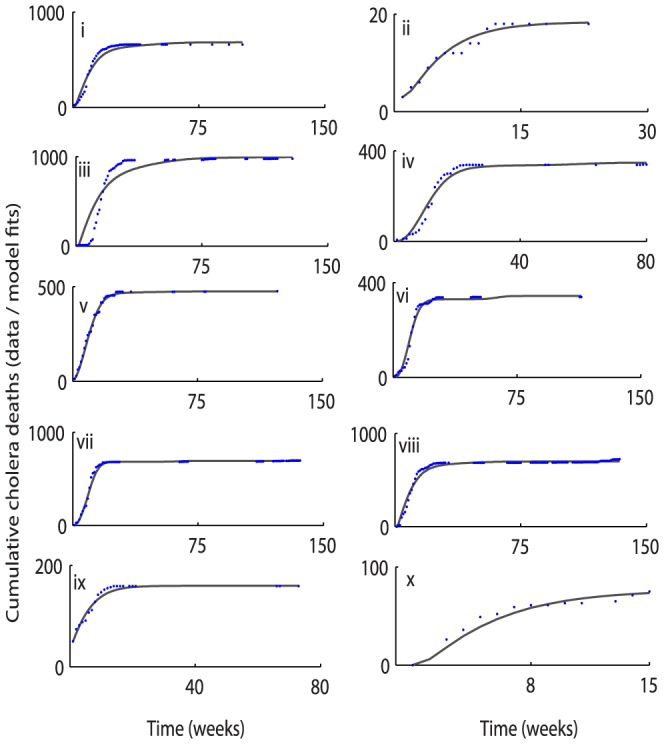
Province-wise cumulative cholera-related deaths. The data points are shown by empty blue circles while the model fits by the solid lines. The plots are given in the same order as of **Figure 3**. The cumulative deaths from the model are plotted using parameter values and initial conditions from **Table S3 and Table S4**.

**Figure 5:**
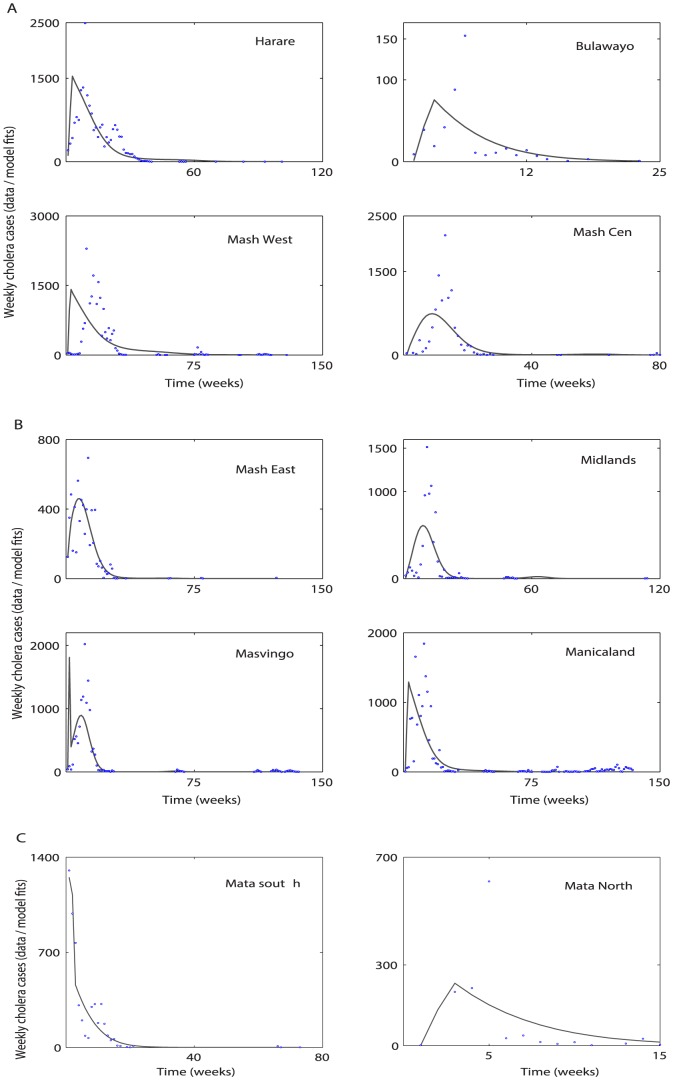
Cholera model fitting for the weekly new cholera cases. The solid line represents the model solution, and blue circles mark the reported cholera cases in the provinces using parameter values and initial conditions from **Table S3 and Table S4**.

**Figure 6:**
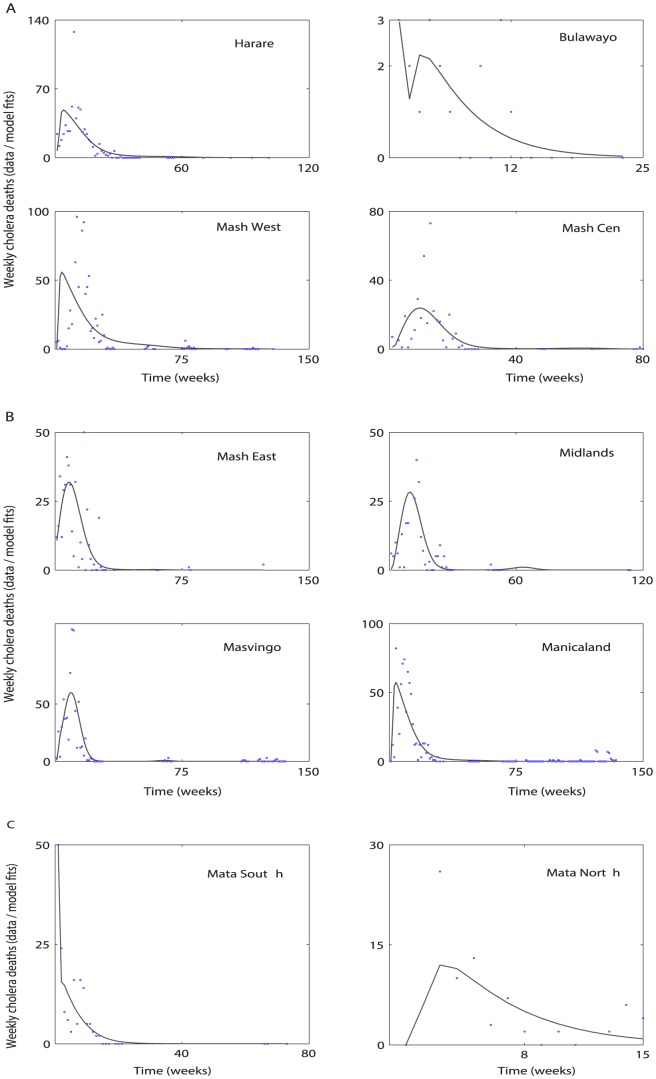
Cholera model fitting for the weekly new cholera deaths. The solid line represents the model solution, and blue circles mark the reported cholera deaths in the provinces using parameter values and initial conditions from **Table S3 and Table S4**.

Cholera model fitting for the cumulative reported cholera cases and deaths are depicted in **Figure 3** and **Figure 4**, respectively. A comparison between weekly reported cholera cases & deaths from each province with the model solution are shown in **Figure 5(A, B, and C)** and **Figure 6(A, B, and C)**, respectively. The estimated model parameters, including human and pathogen demographic parameters, for each province are given in the format [**estimate (95% CI)**], (**Table S3 and Table S4**). Plots for the posterior distributions of the estimated unknown variables of the cholera model (1) are given in **Figure S1 and Figure S2**.

The contributions of low-infectious (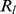) versus high-infectious (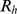) transmission to 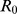 vary widely. Our estimated values of 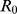, 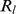 and 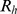 are in good agreement with the previous estimates given by Mukandavire *et. al.* [14], for the provinces Harare, Mashonaland East, Mashonaland Central, Midlands and Masvingo but significantly differ in Bulawayo, Mashonaland West, Manicaland, Matabeleland South and Matabeleland North. In Mashonaland West and Manicaland, contribution of low-infectious (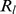) is higher than hyper-infectious (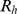) transmission to 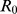 (see Table 3). Opposite trend is observed in the estimates of 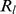 and 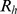 given by Mukandavire *et. al.* [14] in these two provinces. Estimates of 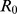 in Bulawayo (0.1022), Matabeleland South (0.7914) and Matabeleland North (0.0541) are all found to be below the unity, which are drastically different from the estimates given by Mukandavire *et. al.* [14].

**Table 3:** Estimates of 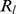, 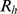 and 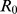.

Zimbabwe province	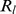	95% CI	% 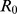	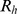	95% CI	% 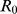	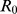	95% CI
Harare	1.2294	1.1885–1.2764	99.06	0.011	0.0004–0.04	0.94	1.2406	1.206–1.285
Bulawayo	0.098	0.0212–0.2083	95.89	0.0042	0–0.025	4.11	0.1022	0.0215–0.2382
Mashonaland West	1.4105	1.338–1.4651	97.66	0.0338	0.0017–0.1387	2.34	1.4443	1.4126–1.4782
Mashonaland Central	0.005	0.0044–0.0057	0.28	1.7701	1.7465–1.8015	99.72	1.775	1.7518–1.8063
Mashonaland East	0.0003	0–0.0015	0.0163	1.8453	1.8387–1.8499	99.98	1.8456	1.8397–1.8501
Midlands	0.0071	0.0059–0.0092	0.39	1.7974	1.7844–1.8101	99.61	1.8045	1.7929–1.8161
Manicaland	1.1146	1.031–1.1575	97.91	0.0238	0.0012–0.1256	2.09	1.1384	1.0982–1.1765
Masvingo	0.0012	1.52E-04 - 0.003	0.065	1.8235	1.8139–1.8350	99.94	1.8246	1.8158–1.8355
Matabeleland North	0.0353	0.0164–0.0875	65.25	0.0188	7.29E-04 - 0.0891	34.75	0.0541	0.0208–0.138
Matabeleland South	0.4925	0.4697–0.5158	62.23	0.2989	0.2499–0.3756	37.77	0.7914	0.74–0.8684

To justify the existence of any seasonal trends in cholera incidence data of different provinces, the values of the test statistic 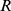, defined in (8), are calculated using weekly data from 18th August, 2008 to 30th June, 2011. The values of 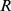 and corresponding p-values for each province are given in Table 4. Significant seasonal trends in cholera incidence data are observed in Harare, Mashonaland West, Mashonaland East, Manicaland and Matabeleland South. Among these five provinces, Harare and Manicaland exhibit highly significant seasonal trend, as confirmed by the corresponding p-value of the test (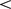 0.01).

**Table 4:** Table showing results for seasonality testing.

Province	Test statistic R	p-value
Harare	9.7992	0.0074 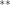
Bulawayo	0.3625	0.8342
Mashonaland West	6.9417	0.0311 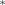
Mashonaland Central	0.1040	0.9493
Mashonaland East	6.1335	0.0466 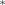
Midlands	0.7608	0.6836
Masvingo	1.1860	0.5527
Manicaland	13.0533	0.0015 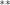
Matabeleland South	7.1850	0.0275 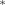
Matabeleland North	0.1395	0.9326

Bold provinces are where the seasonality test result is found to be positive. 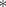: Denote the provinces where seasonality presents at the significance level 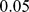 and 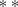: denote the provinces where seasonality present at the significance level 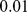.

Our basic model (1) without any interventions, projects 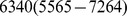 cases and 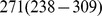 deaths due to cholera in Zimbabwe between the end of epidemic in 2008–09 to January 1, 2012. Table 5 and Table 6 contain the predicted total cholera cases and deaths for the provinces during that time interval. Among the ten provinces, in Mashonaland West and Mashonaland Central the most numbers of cholera cases and deaths are predicted. In Mashonaland West, total 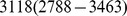 cases, and 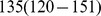 deaths are predicted during the mentioned period. This is about 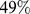 of the total predicted cases and about 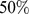 of the total predicted deaths in all provinces of Zimbabwe. In Mashonaland Central, total 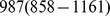 cases, and 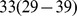 deaths are predicted during the mentioned period. This is about 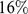 of the total predicted cases and about 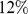 of the total predicted deaths in all provinces of Zimbabwe.

**Table 5:** Number of cases from cholera projected between the end of 2008–09 epidemic to January 1, 2012, by province under base case and under each intervention scenario at an optimal rate.

	Harare	Bulawayo	Mash west	Mash cen	Mash east	Midlands	Masvin	Manica	Mata south	Mata north	Total
**Base cases**	752 (623–936)	5 (4–7)	3118 (2788–3463)	987 (858–1161)	141 (121–163)	163 (131–206)	138 (112–170)	766 (683–859)	188 (173–204)	82 (72–95)	6340 (5565–7264)
**PH & CWD**	111 (96–132)	*	336 (310–381)	50 (39–61)	3 (2–5)	5 (3–8)	2 (1–3)	112 (100–122)	42 (40–44)	30 (25–37)	691 (616–793)
**VA**	699 (198–934)	*	2446 (653–3175)	565 (116–985)	126 (7–159)	141 (7–204)	117 (5–168)	672 (222–858)	170 (79–201)	*	4936 (1287–6684)
**TR**	358 (306–427)	*	1149 (1060–1227)	86 (67–107)	4 (3–6)	6 (4–8)	2 (2–2)	402 (354–440)	126 (120–133)	*	2133 (1916–2350)
**SN**	453 (387–546)	*	1513 (1396–1626)	131 (105–161)	6 (5–9)	10 (8–13)	4 (4–4)	496 (439–546)	146 (138–155)	*	2759 (2482–3060)
**PH & CWD + TR + VA**	102 (71–124)	*	298 (224–324)	50 (39–60)	3 (2–4)	4 (3–6)	1 (1–2)	102 (71–114)	42 (31–47)	31 (25–39)	633 (467–720)
**PH & CWD + TR + SN**	104 (90–123)	*	303 (281–320)	50 (39–61)	3 (2–4)	4 (3–6)	1 (1–2)	105 (94–113)	45 (43–46)	32 (27–39)	647 (580–714)
**PH & CWD + VA + SN**	101 (69–123)	*	287 (213–332)	47 (37–59)	3 (2–5)	5 (3–7)	2 (1–4)	103 (71–116)	40 (29–43)	30 (26–36)	618 (451–725)
**VA + TR + SN**	296 (154–367)	*	991 (544–1075)	77 (58–99)	4 (3–5)	5 (3–8)	2 (2–2)	340 (186–396)	113 (69–127)	80 (60–94)	1908 (1079–2173)
**PH & CWD + TR + VA + SN**	103 (71–123)	*	293 (223–320)	49 (39–60)	3 (2–4)	4 (3–6)	1 (1–2)	101 (71–113)	45 (43–47)	31 (24–39)	630 (477–714)

Data are given in the format [mean (95% CI)].

PH & CWD: Promoting hand-hygiene & clean water distribution; VA: Vaccination; TR: Treatment; SN: Sanitation; PH & CWD + TR + VA: Promoting hand-hygiene & clean water distribution plus treatment with vaccination; PH & CWD + TR + SN: Promoting hand-hygiene & clean water distribution plus treatment with sanitation; PH & CWD + VA + SN: Promoting hand-hygiene & clean water distribution plus vaccination with sanitation; VA + TR + SN: Vaccination plus treatment with sanitation; PH & CWD + TR + VA + SN: Promoting hand-hygiene & clean water distribution plus treatment plus vaccination with sanitation. * indicate the intervention/intervention combination which do not have any effect on case reduction in a province.

**Table:** Table 6. Number of deaths from cholera projected between the end of 2008–09 epidemic to January 1, 2012, by province under base case and under each intervention scenario at an optimal rate.

	Harare	Bulawayo	Mash west	Mash cen	Mash east	Midlands	Masvin	Manica	Mata south	Mata north	Total
**Base deaths**	26 (22–31)	0 (0–0)	135 (120–151)	33 (29–39)	10 (9–12)	8 (6–10)	9 (8–11)	38 (34–42)	6 (5–6)	6 (5–7)	271 (238–309)
**PH & CWD**	5 (4–6)	*	19 (18–21)	3 (2–3)	0 (0–0)	0 (0–0)	0 (0–0)	7 (6–8)	2 (2–2)	3 (2–3)	39 (34–43)
**VA**	24 (8–31)	*	107 (32–138)	19 (5–34)	9 (0–11)	7 (0–10)	8 (0–11)	33 (12–42)	5 (3–6)	*	212 (60–283)
**TR**	8 (7–9)	*	31 (29–33)	3 (2–3)	0 (0–0)	0 (0–0)	0 (0–0)	12 (11–13)	2 (2–3)	*	56 (51–61)
**SN**	16 (14–19)	*	68 (62–73)	5 (4–6)	0 (0–0)	0 (0–1)	0 (0–0)	25 (22–27)	5 (4–5)	*	119 (106–131)
**PH & CWD + TR + VA**	3 (3–4)	*	13 (11–14)	2 (2–3)	0 (0–0)	0 (0–0)	0 (0–0)	5 (4–5)	1 (1–1)	2 (2–3)	26 (23–30)
**PH & CWD + TR + SN**	3 (3–4)	*	13 (12–14)	2 (2–3)	0 (0–0)	0 (0–0)	0 (0–0)	5 (4–5)	1 (1–1)	2 (2–3)	26 (24–30)
**PH & CWD + VA + SN**	5 (3–5)	*	17 (14–19)	3 (2–3)	0 (0–0)	0 (0–0)	0 (0–0)	7 (5–7)	2 (1–2)	3 (2–3)	37 (27–39)
**VA + TR + SN**	7 (4–8)	*	28 (18–30)	2 (2–3)	0 (0–0)	0 (0–0)	0 (0–0)	10 (6–12)	2 (1–2)	4 (3–4)	53 (34–59)
**PH & CWD + TR + VA + SN**	3 (3–4)	*	13 (11–14)	2 (2–3)	0 (0–0)	0 (0–0)	0 (0–0)	5 (4–5)	1 (1–1)	2 (2–3)	26 (23–30)

Data are given in the format [mean (95% CI)].

Notations in the first column are exactly same as Table 5. * indicate the intervention/intervention combination which do not have any effect on death reduction in a province.

To justify the predictive performance of our basic model (1), we compare the predicted cumulative cases and deaths from the end of the epidemic in 2008–2009 to January 1, 2012 with the reported cases and deaths' figures. The reported cumulative cases and deaths during the aforesaid time period are 2225 and 72, respectively [38], which is about 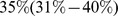 of the model projected cases and about 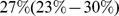 of the model projected deaths. Significant difference in the actual and predicted case and death figures may be attributed to the higher percentage of underreporting of cholera cases and deaths [39], that was not considered while making these predictions. According to WHO, the officially reported cholera cases represent only 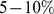 of the actual number of cases those are occurring annually worldwide [39].

We found that, in the African region the countries report cholera cases more consistently than the other countries under WHO [39]. Also Zimbabwe's Integrated Diseases Surveillance & Response Technical guidelines list Cholera among the diseases that must be reported on a daily basis during epidemics to prevent avoidable illness and death [40]. Thus we may expect that the percentage of reported cases is higher in Zimbabwe than the worldwide statistics of under-reporting, although, we do not have the specific figures/numbers from literature depicting the actual percentages of under-reporting in Zimbabwe during the end of epidemic in 2008–09 to January, 1, 2012. The actual reported cases during that period are about 31%–40% of our model predicted cases. Hence this percentage may be considered as an estimate of reporting of cholera cases in Zimbabwe, which is much greater than the worldwide statistics (5%–10%).

It is already mentioned that Mashonaland West and Mashonaland Central are high-risk provinces in terms of cholera incidence between the end of epidemic in 2008–09 and January 1, 2012. Therefore, we discuss the results of different interventions and their layered combinations for these two provinces only.

We have arrived at the following conclusions.

In Mashonaland West, on average 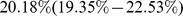 (Table 7) the relative reduction of bacterial ingestion (per week) is observed. By promoting hand-hygiene and clean water supply will avert 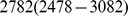 cases (Table 5) and 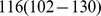 deaths (Table 6). Cost of carrying out this intervention over the period (end of epidemic in 2008–09 to January 1, 2012) is about 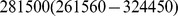 (USD) (Table 8). Cost per averted case in Mashonaland West using hand-hygiene and clean water supply is 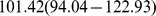 (USD) (Table 9), making this intervention as the cheapest among other single interventions. Again, in spite of total vaccination coverage 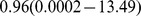 (Table 7), the projected cases and deaths occurred in Mashonaland West will be 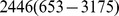 and 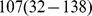, respectively (Tables 5 and 6). Cost per averted case in Mashonaland West using vaccination is 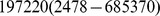 (USD) (Table 9), making this intervention the most costly among other single interventions.

**Table 7:** Average optimal rate at which different intervention should be given between the end of 2008–09 epidemic to January 1, 2012, for each province.

	Harare	Mash west	Mash cen	Mash east	Midlands
**PH & CWD**	12.91(12.16–14.01)	20.18(19.35–22.53)	7.65(6.73–9.63)	3.85(2.37–13.66)	4.26(3.58–7.24)
**VA**	1.17(2.82E-04–14.95)	0.96(2.96E-04–13.49)	1.61(4.46E-04–17.36)	0.96(3.8E-04–13.22)	1.25(2.52E-04–11.74)
**TR**	23.86(22.88–25.33)	30.05(29.49–30.79)	13.91(13.39–15.06)	4.89(3.44–11.76)	9.31(8.81–10.96)
**SN**	10.33(9.11–11.63)	16.66(16.19–17.35)	5.73(5.23–7.13)	2.72(2.57–3.05)	3.82(3.38–5.70)
**PH & CWD + TR + VA**	8.16(4.12–9); 20.32(8.53–22.38); 0.63(2.78E-04–8.11)	11.51(5.95–12.34); 25.11(10.87–27.24); 0.91(3E-04–13.53)	2.41(1.07–3.17); 13.14(4.79–14.48); 0.95(4.39E-04–15.01)	0.30(0.30–0.31); 3.72(1.47–6.45); 1.57(3.8E-04–10.6)	0.31(0.24–0.47); 8.85(2.59–13.52); 0.97(2.52E-04–13.21)
**PH & CWD + TR + SN**	8.40(8.04–8.85); 21.21(19.58–22.01); 0.16(0.15–0.17)	12.05(11.78–12.25); 26.65(26.09–27.39); 0.82(0.74–0.91)	2.49(1.99–3.13); 14(13.43–15.99); 0.15(0.15–0.15)	0.30(0.30–0.30); 4.59(3.26–6.98); 0.0055(0–0.056)	0.32(0.24–0.55); 10.08(8.58–21.71); 0.023(0–0.51)
**PH & CWD + VA + SN**	11.34(5.09–13.48); 1.54(2.82E-04–13.86); 1.53(0.43–1.94)	15.09(7.09–19.40); 2.78(3E-04–13.99); 3.07(0.88–4.80)	7.38(2.18–12.47); 0.75(4.39E-04–14.46); 2.09(0.44–6.77)	3.83(0.90–19.34); 0.73(3.8E-04–11.43); 2.5(0.26–14.59)	4(0.86–8.55); 0.81(2.52E-04–11.3); 2.31(0.09–6.32)
**VA + TR + SN**	1.96(2.82E-04–15.35); 20.24(8.53–23.46); 2.85(0.81–3.55)	0.196(2.95E-04–4.099); 27.85(11.2–29.15); 5.33(1.44–5.63)	1.23(4.39E-04–14.38); 12.66(4.76–14.53); 0.94(0.44–1.26)	0.45(3.8E-04–7.31); 4.31(1.66–7.13); 0.16(0.15–0.20)	1.46(2.52E-04–13.40); 8.4(2.6–13.07); 0.16(0.13–0.19)
**PH & CWD + TR+ VA + SN**	8.25(4.02–8.97); 20.85(8.45–22.45); 0.45(2.39E-04–10.93); 0.16(0.15–0.18)	11.32(5.93–12.23); 24.74(10.85–27.27); 0.84(3E-04–12.03); 0.75(0.21–0.93)	2.41(1.09–3.21); 13.23(4.85–15.61); 0.996(4.39E-04–14.36); 0.15(0.15–0.15)	0.30(0.30–0.30); 4.38(1.83–7.6); 0.51(3.8E-04–12.08); 0.005(0–0.05)	0.31(0.24–0.34); 10.35(8.79–19.02); 2.7E-04(2.5E-04–3.04E-04); 0.002(0–0.039)

	Masvin	Manica	Mata south	Mata north	
**PH & CWD**	4.21(2.37–17.14)	13.33(12.95–13.79)	7.16(7.02–7.37)	6.89(6.59–7.31)	
**VA**	1.65(4.28E-04–16.61)	2.7(2.3E-04–18.8)	1.19(0.001–12.6)	*	
**TR**	4.97(3.54–7.46)	24(23.3–24.7)	13(12.72–13.36)	*	
**SN**	2.64(2.46–3.52)	10.02(9.25–11)	3.67(3.5–3.87)	*	
**PH & CWD + TR + VA**	0.28(0.25–0.30); 4.65(1.47–8.93); 0.91(4.28E-04–11.4)	8.85(4.79–9.6); 20.05(9.18-21.94); 0.49(2.3E-04–5.66)	4.33(2.39–5.05); 11.08(6.17–12.74); 2.02(0.001–15.01)	5.45(4.63–5.90); 11.52(10.02–12.56); 0.19(7.0E-04–2.37)	
**PH & CWD + TR + SN**	0.28(0.25–0.30); 4.94(3.46–8.14); 5.49E-04(0–0.0074)	9.35(9.11–9.56); 21.42(20.93–22.05); 0.22(0.19–0.25)	4.88(4.68–5.06); 12.48(12.16–12.8); 0.024(0.008–0.0415)	5.49(5.19–5.96); 11.6(11.07–12.6); 5.74(0–7.21)	
**PH & CWD + VA + SN**	4.24(0.498–17.86); 1.22(4.28E-04–11.6); 2.70(0.48–11.85)	12.18(5.75–13.34); 0.83(2.3E-04-11.75); 2.0(0.53–2.39)	6.34(3.24–7.3); 1.59(0.001–13.42); 0.20(0.11–0.27)	6.87(6.58–7.38); 9.6E-04(6.5E-04–1.1E-03); 4.14(0–5.18)	
**VA + TR + SN**	1.01(4.28E-04–11.5); 4.51(1.46–6.42); 0.148(0.148–0.149)	1.51(2.3E-04–15.14); 21.09(9.32-23.47); 3.32(0.99–3.79)	1.37(0.001–13.72); 11.92(6.22–13.14); 1.02(0.25–1.3)	0.036(7E-04–0.44); 11.57(10.06–12.57); 0.0018(0–0.033)	
**PH & CWD + TR + VA + SN**	0.28(0.25–0.30); 5.09(1.51–10.54); 0.51(4.28E-04–12.07); 6.37E-04(0–0.01)	8.79(4.70–9.54); 19.91(9.13–21.9); 0.87(0.0002–12.57); 0.21(0.15–0.25)	4.88(4.67–5.06); 12.47(12.17–12.79); 0.001(0.001–0.001); 0.023(0.008–0.0409)	5.4(2.9–6.0); 11.4(6.7–12.6); 0.53(6E-04-12.59); 1E-04(0-2E-03)	

Data for vaccination are given according to its total coverage percentage and data for treatment, hand-hygiene & clean water distribution (PH & CWD) and sanitation given according to average coverage percentage per day. All data are given in the format [mean (95% CI)]. Data for the Bulawayo province is not given as different interventions have no effect on case or death reduction in this region.

Notations in the first column are exactly same as Table 5. * indicate the intervention/intervention combinations which do not have any effect on case or death reduction in a province.

**Table 8:** Optimal cost (in USD) projected between the end of 2008–09 epidemic to January 1, 2012, by province under each intervention scenario at an optimal rate.

	Harare	Mash west	Mash cen	Mash east	Midlands
**PH & CWD**	8.28*E*+04 (7.39*E*+04–9.56*E*+04)	2.82*E*+05 (2.62*E*+05–3.24*E*+05)	3.75*E*+04 (3.14*E*+04–4.36*E*+04)	8.16*E*+03 (6.28*E*+03–1.89*E*+04)	8.95*E*+03 (7.43*E*+03–1.39 *E*+04)
**VA**	3.25*E*+06 (4.12*E*+05–3.20*E*+07)	4.55*E*+06 (2.28*E*+06–2.34*E*+07)	3.20*E*+06 (5.89*E*+05–1.85*E*+07)	1.91*E*+06 (1.68*E*+05–1.87*E*+07)	4.07*E*+06 (1.27*E*+05–3.06*E*+07)
**TR**	1.26*E*+05 (1.10*E*+05–1.48*E*+05)	5.13*E*+05 (4.76*E*+05–5.48*E*+05)	2.78*E*+04 (2.19*E*+04–3.42*E*+04)	2.69*E*+03 (2.17*E*+03–4.40*E*+03)	3.07*E*+03 (2.29*E*+03–3.98*E*+03)
**SN**	2.96*E*+05 (2.57*E*+05–3.49*E*+05)	1.24*E*+06 (1.15*E*+06–1.34*E*+06)	8.05*E*+04 (6.55*E*+04–9.65*E*+04)	9.77*E*+03 (8.34*E*+03–1.36*E*+04)	1.16*E*+04 (9.49*E*+03–1.40*E*+04)
**PH & CWD + TR + VA**	1.99*E*+06 (4.48*E*+04–2.67*E*+07)	2.22*E*+06 (1.49*E*+05–2.16*E*+07)	1.25*E*+06 (1.78*E*+04–1.75*E*+07)	3.91*E*+06 (2.31*E*+03–1.83*E*+07)	3.17*E*+06 (2.57*E*+03–2.97*E*+07)
**PH & CWD + TR + SN**	4.97*E*+04 (4.45*E*+04–5.74*E*+04)	1.58*E*+05 (1.48*E*+05–1.65*E*+05)	2.20*E*+04 (1.78*E*+04–2.63*E*+04)	2.74*E*+03 (2.31*E*+03–4.03*E*+03)	3.23*E*+03 (2.41*E*+03–4.29*E*+03)
**PH & CWD + VA + SN**	3.99*E*+06 (7.20*E*+04–3.21*E*+07)	6.50*E*+06 (2.49*E*+05–2.46*E*+07)	1.20*E*+06 (3.21*E*+04–1.76*E*+07)	1.60*E*+06 (6.32*E*+03–1.82*E*+07)	2.59*E*+06 (7.99*E*+03–3.12*E*+07)
**VA + TR + SN**	4.55*E*+06 (1.02*E*+05–3.29*E*+07)	1.09*E*+06 (4.21*E*+05–1.60*E*+07)	1.98*E*+06 (2.02*E*+04–1.78*E*+07)	9.65*E*+05 (2.15*E*+03–1.61*E*+07)	4.31*E*+06 (2.45*E*+03–3.24*E*+07)
**PH & CWD + TR+ VA + SN**	1.11*E*+06 (4.47*E*+04–2.52*E*+07)	2.36*E*+06 (1.48*E*+05–2.11*E*+07)	1.31*E*+06 (1.78*E*+04–1.74*E*+07)	7.55*E*+05 (2.31*E*+03–1.84*E*+07)	3.21*E*+03 (2.41*E*+03–4.34*E*+03)

	Masvin	Manica	Mata south	Mata north	Total
**PH & CWD**	6.96*E*+03 (5.02*E*+03–2.05*E*+04)	1.15*E*+05 (1.06*E*+05–1.24*E*+05)	3.23*E*+04 (3.03*E*+04–3.43*E*+04)	4.26*E*+04 (3.82*E*+04–4.97*E*+04)	6.16*E*+05 (5.60*E*+05–7.25*E*+05)
**VA**	2.54*E*+06 (1.53*E*+05–1.90*E*+07)	6.04*E*+06 (6.38*E*+05–3.75*E*+07)	1.03*E*+06 (9.39*E*+04–7.21*E*+06)	*	2.66*E*+07 (4.46*E*+06–1.87*E*+08)
**TR**	1.53*E*+03 (1.39*E*+03–1.72*E*+03)	2.03*E*+05 (1.83*E*+05–2.22*E*+05)	3.87*E*+04 (3.52*E*+04–4.19*E*+04)	*	9.16*E*+05 (8.31*E*+05–1.00*E*+06)
**SN**	6.84*E*+03 (6.32*E*+03–7.67*E*+03)	4.63*E*+05 (4.18*E*+05–5.10*E*+05)	8.29*E*+04 (7.62*E*+04–9.07*E*+04)	*	2.19*E*+06 (1.99*E*+06–2.42*E*+06)
**PH & CWD + TR + VA**	1.61*E*+06 (1.56*E*+03–1.72*E*+07)	2.66*E*+06 (6.34*E*+04–2.32*E*+07)	1.36*E*+06 (2.04*E*+04–7.55*E*+06)	2.56*E*+05 (2.71*E*+04–2.85*E*+06)	1.84*E*+07 (3.29*E*+05–1.64*E*+08)
**PH & CWD + TR + SN**	1.71*E*+03 (1.56*E*+03–1.87*E*+03)	6.77*E*+04 (6.31*E*+04–7.25*E*+04)	2.17*E*+04 (2.04*E*+04–2.29*E*+04)	3.02*E*+04 (2.71*E*+04–3.51*E*+04)	3.57*E*+05 (3.27*E+05–3.90E*+05)
**PH & CWD + VA + SN**	2.23*E*+06 (5.23*E*+03–1.75*E*+07)	2.87*E*+06 (1.03*E*+05–2.48*E*+07)	1.25*E*+06 (3.02*E*+04–7.07*E*+06)	4.25*E*+04 (3.81*E*+04–4.96*E*+04)	2.23*E*+07 (5.44*E* +05–1.73*E*+08)
**VA + TR + SN**	1.89*E*+06 (1.42*E*+03–1.75*E*+07)	3.91*E*+06 (1.69*E*+05–3.44*E*+07)	9.29*E*+05 (3.45*E*+04–7.21*E*+06)	1.86*E*+05 (4.69*E*+04–1.72*E*+06)	1.98*E*+07 (8.00*E*+05–1.76*E*+08)
**PH & CWD + TR + VA + SN**	7.20*E*+05 (1.57*E*+03–1.76*E*+07)	2.98*E*+06 (6.33*E*+04–2.62*E*+07)	2.17*E*+04 (2.04*E*+04–2.29*E*+04)	4.71*E*+05 (2.71*E*+04–1.04*E*+07)	9.74*E*+06 (3.27*E*+05–1.36*E*+08)

Costs are given in the format [mean(95% CI)]. Cost corresponding to Bulawayo province is not given as different interventions have no effect on case or death reduction in this region.

Notations in the first column are exactly same as Table 5. Here *Ek* = 10*^k^*. * indicate the intervention/intervention combination which do not have any effect on case or death reduction in a province.

**Table 9:** Cost per averted case (in USD) projected between the end of 2008–09 epidemic to January 1, 2012, by province under each intervention scenario at an optimal rate.

	Harare	Mash west	Mash cen	Mash east	Midlands
**PH & CWD**	1.30*E*+02 (1.17*E*+02–1.42*E*+02)	1.01*E*+02 (9.40*E*+01–1.23*E*+02)	4.02*E*+01 (3.35*E*+01–4.59*E*+01)	5.92*E*+01 (4.70*E*+01–1.31*E*+02)	5.73*E*+01 (4.97*E*+01–9.63 *E*+01)
**VA**	5.30*E*+05 (4.73*E*+04–8.11*E*+05)	1.97*E*+05 (2.48*E*+03–6.85*E*+05)	1.79*E*+04 (1.99*E*+03–1.07*E*+05)	1.26*E*+05 (9.88*E*+04–1.55*E*+05)	1.78*E*+05 (1.17*E*+05–2.19*E+05)*
***TR***	*3.22E*+02 (2.89*E*+02–3.52*E*+02)	2.62*E*+02 (2.42*E*+02–2.82*E*+02)	3.09*E*+01 (2.41*E*+01–3.68*E*+01)	1.95*E*+01 (1.74*E*+01–2.76*E*+01)	1.95*E*+01 (1.71*E*+01–2.24*E+01)*
***SN***	*9.99E*+02 (8.96*E*+02–1.09*E*+03)	7.77*E*+02 (7.21*E*+02–8.41*E*+02)	9.44*E*+01 (7.42*E*+01–1.12*E*+02)	7.24*E*+01 (6.66*E*+01–8.84*E*+01)	7.65*E*+01 (7.01*E*+01–8.54*E+01)*
***PH & CWD + TR + VA***	*3.12E*+03 (6.89*E*+01–4.16*E*+04)	7.87*E*+02 (5.25*E*+01–7.66*E*+03)	1.42*E*+03 (1.91*E*+01–2.06*E*+04)	2.80*E*+04 (1.81*E*+01–1.35*E*+05)	2.16*E*+04 (1.82*E+01–2.10E*+05)
**PH & CWD + TR + SN**	7.70*E*+01 (6.87*E*+01–8.40*E*+01)	5.61*E*+01 (5.20*E*+01–5.99*E*+01)	2.36*E*+01 (1.91*E*+01–2.74*E*+01)	1.98*E*+01 (1.79*E*+01–2.53*E*+01)	2.04*E*+01 (1.80*E+01–2.61E*+01)
**PH & CWD + VA + SN**	6.03*E*+03 (1.15*E*+02–4.96*E*+04)	2.25*E*+03 (8.86*E*+01–8.71*E*+03)	1.36*E*+03 (3.24*E*+01–2.04*E*+04)	1.12*E*+04 (4.55*E*+01–1.28*E*+05)	1.80*E*+04 (5.19*E+01–2.03E*+05)
**VA + TR + SN**	8.25*E*+03 (2.45*E*+02–6.00*E*+04)	4.72*E*+02 (2.00*E*+02–6.39*E*+03)	2.23*E*+03 (2.25*E*+01–2.09*E*+04)	6.60*E*+03 (1.70*E*+01–1.12*E*+05)	2.94*E*+04 (1.75*E*+01–2.24*E*+05)
**PH & CWD + TR + VA + SN**	1.87*E*+03 (6.86*E*+01–4.38*E*+04)	8.34*E*+02 (5.20*E*+01–7.53*E*+03)	1.50*E*+03 (1.91*E*+01–2.04*E*+04)	5.07*E*+03 (1.80*E*+01–1.25*E*+05)	2.02*E*+01 (1.78E+01–2.41*E*+01)

	Masvin	Manica	Mata south	Mata north	
**PH & CWD**	5.18*E*+01 (3.31*E*+01–1.54*E*+02)	1.76*E*+02 (1.67*E*+02–1.88*E*+02)	2.22*E*+02 (2.06*E*+02–2.34*E*+02)	8.18*E*+02 (7.26*E*+02–9.06*E*+02)	
**VA**	1.19*E*+05 (9.24*E*+04–1.39*E*+05)	1.05*E*+06 (4.93*E*+04–1.50*E*+06)	1.86*E*+05 (4.10*E*+04–2.49*E*+05)	*	
**TR**	1.14*E*+01 (9.66*E*+00–1.32*E*+01)	5.59*E*+02 (5.19*E*+02–6.02*E*+02)	6.29*E*+02 (5.21*E*+02–6.95*E*+02)	*	
**SN**	5.16*E*+01 (4.42*E*+01–5.93*E*+01)	1.72*E*+03 (1.60*E*+03–1.86*E*+03)	2.00*E*+03 (1.66*E*+03–2.21*E*+03)	*	
**PH & CWD + TR + VA**	1.12*E*+04 (1.08*E*+01–1.24*E*+05)	3.90*E*+03 (9.69*E*+01–3.30*E*+04)	8.47*E*+03 (1.40*E*+02–4.92*E*+04)	4.21*E*+03 (5.45*E*+02–4.57*E*+04)	
**PH & CWD + TR + SN**	1.27*E*+01 (1.08*E*+01–1.43*E*+01)	1.03*E*+02 (9.66*E*+01–1.10*E*+02)	1.52*E*+02 (1.40*E*+02–1.61*E*+02)	6.04*E*+02 (5.37*E*+02–6.66*E*+02)	
**PH & CWD + VA + SN**	1.56*E*+04 (3.56*E*+01–1.25*E*+05)	4.23*E*+03 (1.60*E*+02–3.74*E*+04)	7.68*E*+03 (2.04*E*+02–4.43*E*+04)	8.17*E*+02 (7.26*E*+02–9.05*E*+02)	
**VA + TR + SN**	1.36*E*+04 (9.84*E*+00–1.24*E*+05)	7.10*E*+03 (4.27*E*+02–6.01*E*+04)	7.92*E*+03 (4.67*E*+02–6.02*E*+04)	4.56*E*+04 (1.22*E*+04–1.12*E*+05)	
**PH & CWD + TR + VA + SN**	4.84*E*+03 (1.08*E*+01–1.20*E*+05)	4.37*E*+03 (9.66*E*+01–3.85*E*+04)	1.52*E*+02 (1.40*E*+02–1.61*E*+02)	8.01*E*+03 (5.46*E*+02–1.79*E*+05)	

Data are given in the format [mean (95% CI)]. Data corresponding to Bulawayo province is not given as different interventions have no effect on case or death reduction in this region.

Notations in the first column are exactly same as Table 5. Here *Ek* = 10*^k^*. * indicate the intervention/intervention combination which do not have any effect on case or death reduction in a province.

Among layered interventions in Mashonaland West, hand-hygiene & clean water distribution with treatment and sanitation is the most cost-effective and will avert 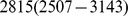 cases (Table 5) and 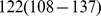 deaths (Table 6). Cost per averted case using this intervention is 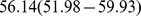 (USD) (Table 9). Hand-hygiene & clean water distribution with vaccination and sanitation is the most costly intervention in Mashonaland West with cost per averted case is 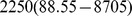 (USD) (Table 9).

For Mashonaland Central, among single interventions, hand-hygiene & clean water distribution will avert most numbers of cases and deaths. Projected cases and deaths using hand-hygiene & clean water distributions are 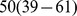 and 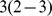, respectively (Tables 5 and 6). Cost per averted case using this intervention is 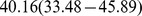
(USD) (Table 9). Treatment is found to be the cheapest among other single interventions in Mashonaland Central with cost per averted case being 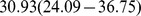
(USD) (Table 9). Treatment of average 13.91% (13.39%–15.06%) (Table 7) cholera infected individuals (per week) will avert 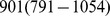 cases (Table 5) and 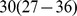 deaths (Table 6), respectively.

Among layered interventions in Mashonaland Central, hand-hygiene & clean water distribution with vaccination and sanitation will avert most numbers of cases and deaths. Projected cases and deaths using this layered intervention are 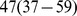 and 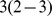, respectively (Tables 5 and 6). Cost per averted case using this intervention is 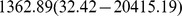 (USD) (Table 9). In terms of cost effectiveness hand-hygiene & clean water distribution with treatment and sanitation is found to be the cheapest layered intervention with cost per averted case being 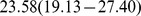 (USD) (Table 9). This intervention combination will avert 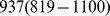 cases and 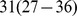 deaths, respectively (Tables 5 and 6).

## Discussion

Our analysis suggests that, the routes of cholera transmission vary from province to province, that agrees with the findings of Mukandavire *et. al.* [14]. This heterogeneity in transmission dynamics may be due to the diverse geographic and climatic conditions across the country. A similar pattern in transmission dynamics is observed in Harare, Mashonaland West, Manicaland and Matabeleland South, where seasonality in cholera incidence was observed. In these provinces, slow transmission route is a dominating factor (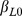 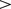 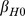) for cholera transmission (**Table S3**). Earlier studies on cholera suggest that slow transmission route is more correlated to the climatic and environmental factors [17], [18], [41]–[47] and is the main cause for seasonal dynamics of cholera [41], [48]–[51]. Unfortunately, due to lack of climatic data of Zimbabwe, we are unable to draw any quantitative inference, for example, whether inter-annual climatic variation in different provinces affects the transmission dynamics of cholera or not.

Estimate of human shedding rate (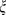) (**Table S3**) in Bulawayo province differs from other nine provinces (one order of magnitude higher than for the other provinces). A possible reason for such difference may be due to the fact that the city receives portable water supply from five surface dams [52] and constantly suffers from improper waste management system [53]. In spite of this; the national water supply agency of Zimbabwe (ZINWA) is not in charge in supplying water in Bulawayo [54].

Mukandavire *et. al.* [14] estimated 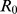 for *2008*–*2009* cholera epidemics in Zimbabwe with constant transmission rate but to our knowledge, this is the first time that the basic reproduction numbers with periodic transmission rate are estimated for cholera epidemics in Zimbabwe or any other country. It is also to be noted that our data set contains much longer time scale (from the beginning of cholera epidemic in *2008–2009* to *June 2011*) than the earlier analyzed data [14]. It is already pointed out that the estimated values of 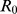 in Bulawayo, Mashonaland West, Manicaland, Matabeleland South and Matabeleland North by Mukandavire *et. al.* [14] differ with our estimates. The disease dynamics of cholera may not be captured properly by assuming constant contact rate between human and bacterial populations over time, as it also depends on temporal forcing. Thus, the model with periodic environment is more appropriate than the previous studies. We believe that the prediction, thereby proposed, will be helpful for policy makers.

Optimal cost effective study in Zimbabwe, from the end of epidemic in 2008–09 to January 1, 2012, suggests that, as a single intervention hand-hygiene & clean water supply is the most cost-effective way to control a future cholera outbreak in those regions where slow transmission is the dominating factor for cholera transmission (see, Tables 8 and 9). In terms of cases and deaths reduction during epidemic hand-hygiene & clean water supply is by far the most effective individual intervention among the other single interventions (see Tables 5 and 6). This result is in good agreement with the observations by Andrews and Basu [6], where they argued that hand-hygiene & clean water distributions will avert more cases and deaths than treatment and vaccination during the epidemic in Haiti. Treatment is the most cost-effective in those regions where hyper-infectious transmission is the main factor for cholera transmission (see, Tables 8 and 9). This result is in well agreement with the previous observation of Naficy *et. al.* [55] on the control of cholera in sub-Saharan refugee settings. Treatment and hand-hygiene & clean water supply with any other intervention combination will also be cost-effective, which could avert thousands of cases and hundreds of deaths in Zimbabwe at a minimal cost. A synchronized, timely and efficient intervention will effectively reduce the severity of the disease and number of deaths. Our mathematical model and its prediction will help the public health authority of Zimbabwe for making suitable intervention strategies. We also believe that, a similar method can be applied for endemic and epidemic cholera outbreaks in other regions/countries as well, in particular, with seasonal patterns in disease transmission.

## Supporting Information

Figure S1 **Marginal distributions of the parameters of the cholera model (1) for different provinces.**
(PDF)

Figure S2 **Marginal distributions of the initial demographic variables of the cholera model (1) for different provinces.**
(PDF)

Table S1 **Definition of the cholera model (1) parameters and their base values.**
(PDF)

Table S2 **Definition of the cholera intervention model (13) parameters and their minimum and maximum values.**
(PDF)

Table S3 **Estimated parameters of the cholera model (1).** All data are given in the format [estimate (95% CI)].
(PDF)

Table S4 **Estimated initial demographic variables of the cholera model (1).** All data are given in the format [estimate (95% CI)].
(PDF)

Appendix S1
**Details on the mathematical stability analysis of the cholera model (1).** Details on the estimation procedure of the basic reproduction number (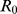) in periodic environment. Details on the intervention cost optimization procedure.(PDF)
