# Supplementary figures and images for: An Optimal Cost Effectiveness Study on Zimbabwe Cholera Seasonal Data from 2008–2011
Source: PLoS One. 2013 Dec 3;8(12):e81231. doi: 10.1371/journal.pone.0081231 (PMC3849194; doi:10.1371/journal.pone.0081231)

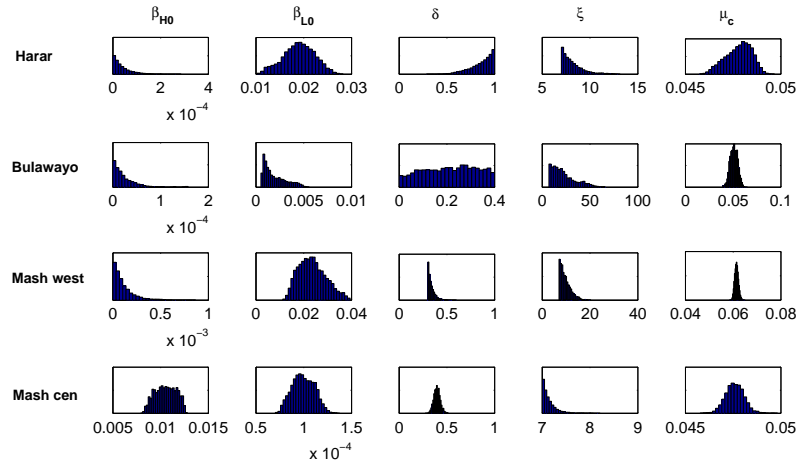

(a)

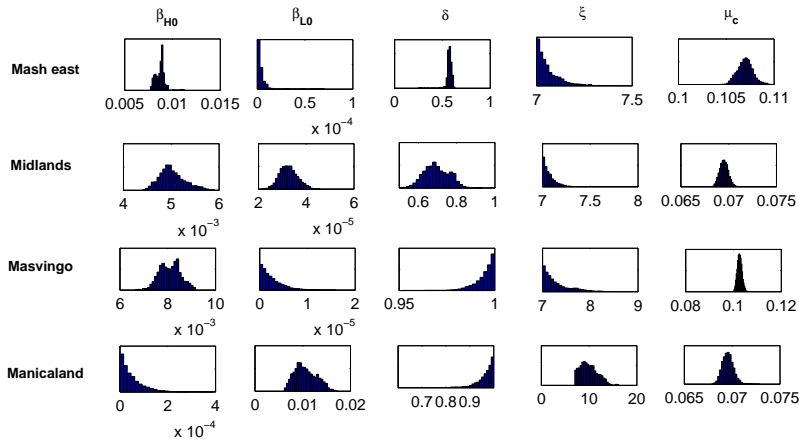

(b)

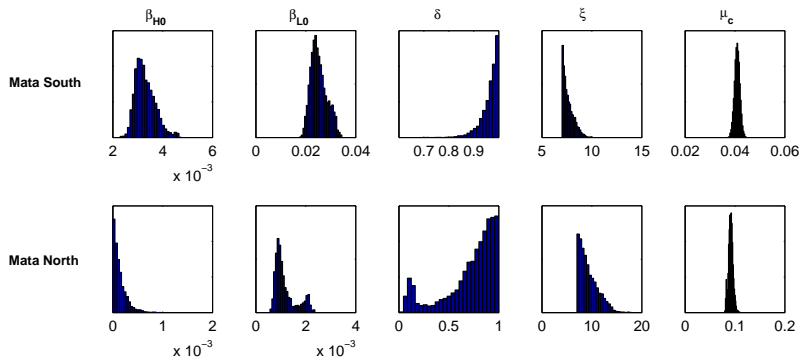

(c)

Supplement: Figure S1 — Marginal distributions of the parameters of the cholera model (1) for different provinces. (PDF) [file pone.0081231.s001.pdf]

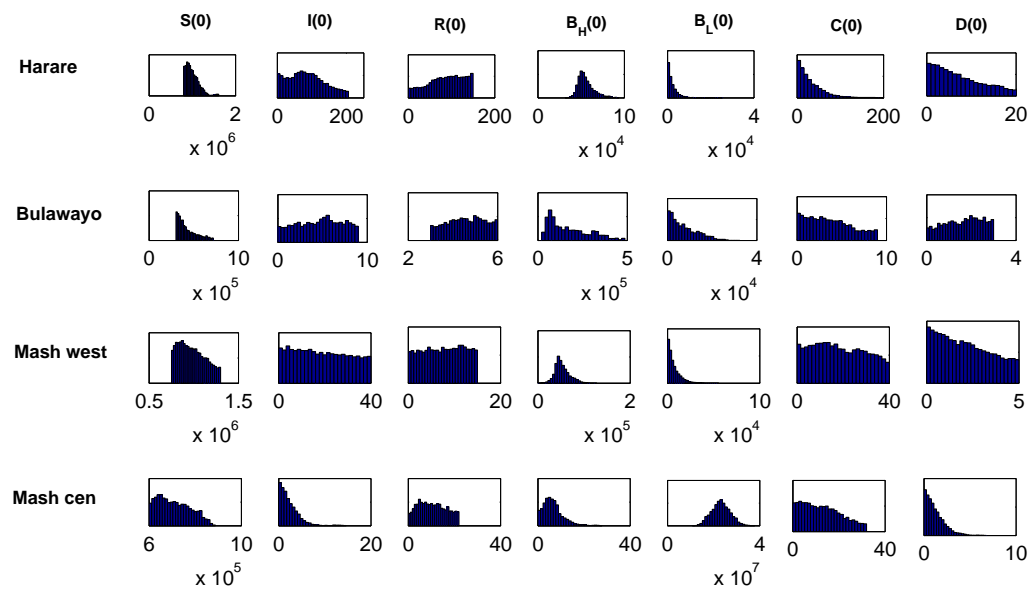

(a)

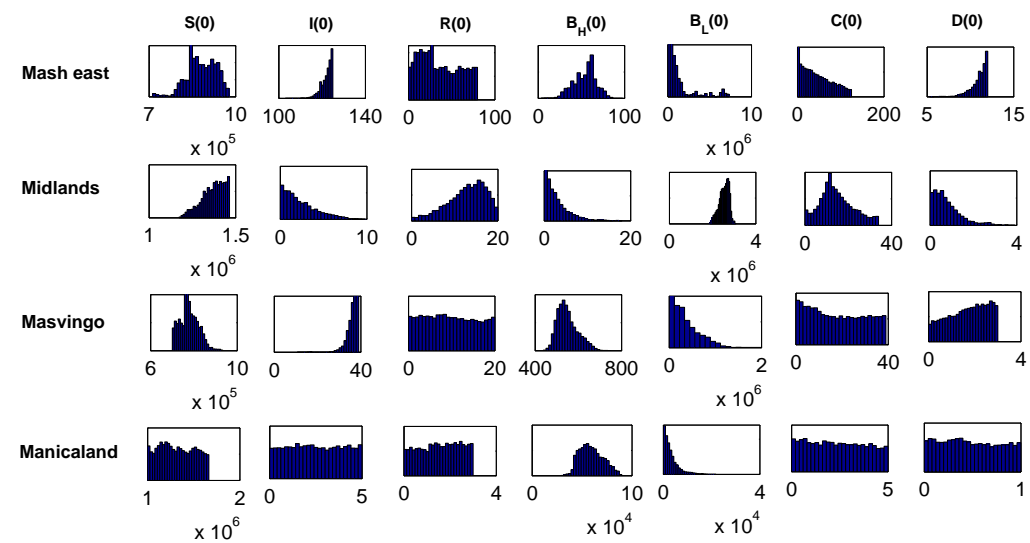

(b)

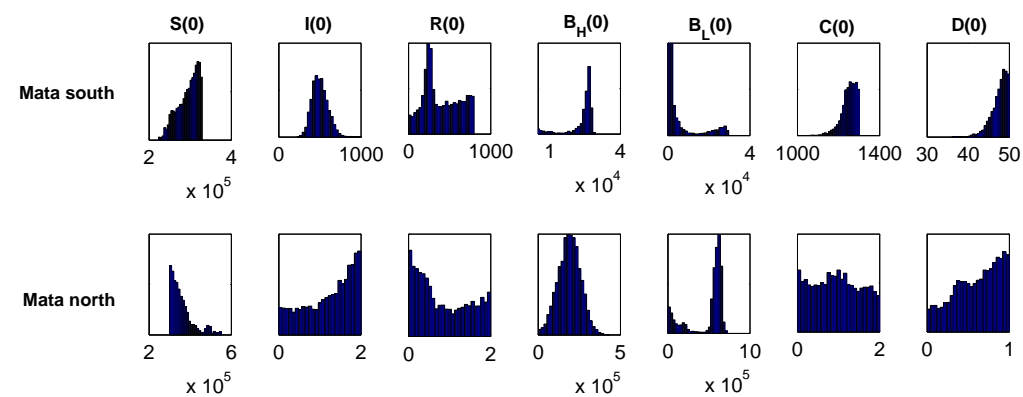

(c)

Supplement: Figure S2 — Marginal distributions of the initial demographic variables of the cholera model (1) for different provinces. (PDF) [file pone.0081231.s002.pdf]
