# Supplementary material for: An Optimal Cost Effectiveness Study on Zimbabwe Cholera Seasonal Data from 2008–2011
Source: PLoS One. 2013 Dec 3;8(12):e81231. doi: 10.1371/journal.pone.0081231 (PMC3849194; doi:10.1371/journal.pone.0081231)
Supplement: Table S1 — Definition of the cholera model (1) parameters and their base values. (PDF) [file pone.0081231.s003.pdf]

Parameter	Symbol	Value	Reference
Birth rate of human (Zimbabwe)	μ_b	$6.02 \cdot 10^{-4}$ week^{-1}	[1]
Death rate of human (Zimbabwe)	μ_d	$3.08 \cdot 10^{-4}$ week^{-1}	[1]
Recruitment rate of human	Π_H	$\mu_b \cdot (S(0) + I(0) + R(0))$ week^{-1}	
Rate of loss of natural immunity	ω	0.0238 week^{-1}	[2, 3, 4]
Minimum transmission rate of hyper-infectious Bacterium	β_{H0}	Estimated	
Minimum transmission rate of low-infectious Bacterium	β_{L0}	Estimated	
Amplitude of seasonality	δ	Estimated	
Half saturation constant (low-infectious bacterium)	K_L	10^6 cells \ ml	[5, 6]
Half saturation constant (high-infectious bacterium)	K_H	$K_L \div 700$ cells \ ml	[7, 6]
Natural recovery rate of human	γ	1.4 week^{-1}	[8, 9]
Death due to cholera infection	μ_c	Estimated	
Rate of contribution to HI *V. cholerae* in aquatic environment	ξ	Estimated	
Rate of decay from hyper-infectious to low-infectious bacterium	χ	33.6 week^{-1}	[7]
Net death rate of non-HI vibrios in the environment	δ_L	$\frac{7}{30}$ week^{-1}	[6, 10, 11]

References

[1] Central Intelligence Agency (2009), *The World Factbook*. Available: https://www.cia.gov/library/publications/download/download-2009/index.html. Accessed July 10, 2012.

[2] King AA, Ionides EL, Pascual M, Bouma MJ (2008) Inapparent infections and cholera dynamics. Nature 454(7206): 877 - 880.

[3] Levine MM, Black RE, Clements ML, Cisneros L, Nalin DR, et al (1981) Duration of Infection-Derived Immunity to Cholera. J Infect Dis 143(6): 818-20.

[4] Woodward WE (1971) Cholera Reinfection in Man. J Infect Dis 123(1): 61-66.

[5] Codeço CT (2001) Endemic and epidemic dynamics of cholera: the role of the aquatic reservoir. BMC Infect Dis: 1:1.

[6] Hartley DM, Morris JGJr, Smith DL (2006) Hyperinfectivity: A Critical Element in the Ability of V. cholerae to Cause Epidemics? PLoS Med 3(1): 63 - 69.

[7] Merrell DS, Butler SM, Qadri F, Dolganov NA, Alam A (2002) Host-induced epidemic spread of the cholera bacterium. Nature 417: 642 - 645.

[8] Rahaman MM, Majid MA, Alam AKMJ, Islam MR (1976) Effects of doxycycline in actively purging cholera patients: a double-blind clinical trial. Antimicrob Agent Chem 10(4): 610 - 612.

[9] Levine MM, Kaper JB, Herrington D, Losonsky G, Morris JG, et al (1981) Volunteer studies of deletion mutants of Vibrio cholerae O1 prepared by recombinant techniques. Infect Immun 56(1): 161-67.

[10] Kaper JB, Morris JG, Levine MM (1995) Cholera. Clin Micro Rev 8: 48 - 86.

[11] Tudor V, Strati I (1977) Smallpox, cholera. Abacus Press, Tunbridge Wells(England).
