# Supplementary material for: An Optimal Cost Effectiveness Study on Zimbabwe Cholera Seasonal Data from 2008–2011
Source: PLoS One. 2013 Dec 3;8(12):e81231. doi: 10.1371/journal.pone.0081231 (PMC3849194; doi:10.1371/journal.pone.0081231)
Supplement: Table S2 — Definition of the cholera intervention model (13) parameters and their minimum and maximum values. (PDF) [file pone.0081231.s004.pdf]

**Parameter**	**Symbol**	**Minimum value**	**Maximum value**	**Reference**
Relative rate of reduction in bacterial ingestion rate by promoting Hand-hygiene and clean water supply.	$\theta(t)$	0 week^{-1}	0.8 week^{-1}	[1]
Rate of Vaccination	$p(t)$	0 week^{-1}	0.35 week^{-1}	[2]
Vaccine efficiency	σ	0.67	0.67	[3, 4, 5]
Rate of waning, vaccine induced immunity	ϵ	0.5 year^{-1}	0.5 year^{-1}	[3, 4, 5]
Proportion of infected individuals receiving antibiotics/oral-rehydration therapy	$\alpha(t)$	0 week^{-1}	0.7 week^{-1}	[2]
Relative rate of recovery, receiving antibiotics/oral-rehydration therapy	λ	2.3	2.3	[6, 7, 3]
Relative rate of shedding, receiving antibiotics/oral-rehydration therapy	ψ	0.52	0.52	[6, 7, 3]
Relative rate of reduction in shedding by promoting sanitation	$s(t)$	0 week^{-1}	0.4 week^{-1}	[1]

References

[1] World Health Organization (2009), *Global Task Force on Cholera Control, Cholera country profile: Zimbabwe*. Available: http://www.who.int/cholera/countries/en/Zimbabwe/. Accessed October 6, 2012.

[2] Neilan RLM, Schaefer E, Gaff H, Fister KR, (2010) Modeling Optimal Intervention Strategies for Cholera. Bull Math Biol 72(8): 2004 - 2018.

[3] Andrews JR, Basu S, (2011) Transmission Dynamics and Control of Cholera in Haiti: An Epidemic Model. Lancet 377(9773): 1248 - 1255.

[4] Sur D, Lopez AL, Kanungo S (2009) Efficacy and safety of a modified killed-whole-cell oral cholera vaccine in India: an interim analysis of a cluster-randomized, double-blind, placebo-controlled trial. Lancet 374(9702): 1694 - 1702.

[5] Thiem VD, Deen JL, von Seidlein L (2006) Long-term effectiveness against cholera of oral killed whole-cell vaccine produced in Vietnam. Vaccine 24(20): 4297-4303.

[6] Rahaman MM, Majid MA, Alam AKMJ, Islam MR (1976) Effects of doxycycline in actively purging cholera patients: a double-blind clinical trial. Antimicrob Agent Chem 10(4): 610 - 612.

[7] Saha D, Karim MM, Khan WA (2006) Single-Dose Azithromycin for the Threatment of Cholera in Adults. N Engl J Med 354(23): 2452 - 2462.
