# Supplementary material for: An Optimal Cost Effectiveness Study on Zimbabwe Cholera Seasonal Data from 2008–2011
Source: PLoS One. 2013 Dec 3;8(12):e81231. doi: 10.1371/journal.pone.0081231 (PMC3849194; doi:10.1371/journal.pone.0081231)
Supplement: Table S3 — Estimated parameters of the cholera model (1). All data are given in the format [estimate (95% CI)]. (PDF) [file pone.0081231.s005.pdf]

| Zimbabwe province | $\beta_{H0}(\text{week}^{-1})$ | $\beta_{L0}(\text{week}^{-1})$ | $\delta$ | $\xi\left(\frac{\text{cells}}{\text{mL}\cdot\text{person}\cdot\text{week}}\right)$ | $\mu_c(\text{week}^{-1})$ |
|---|---|---|---|---|---|
| Harare | $9.973\cdot E-5$ <br> $(0.096-14.1)\cdot E-5$ | 0.017 <br> (0.012−0.025) | 0.923 <br> (0.574−0.996) | 9.376 <br> (7.03−11.32) | 0.048 <br> (0.046−0.049) |
| Bulawayo | $4.97\cdot E-6$ <br> $(0.552-90.2)\cdot E-6$ | $8.26\cdot E-4$ <br> $(6.42-46.4)\cdot E-4$ | 0.216 <br> (0.015−0.389) | 18.1 <br> (7.56−51.56) | 0.0455 <br> (0.0433−0.0584) |
| Mashonaland West | $8.518\cdot E-5$ <br> $(0.30-43.5)\cdot E-5$ | 0.022 <br> (0.014−0.036) | 0.32 <br> (0.30−0.44) | 9.87 <br> (7.09−15.38) | 0.061 <br> (0.060−0.063) |
| Mashonaland Central | $9.59\cdot E-3$ <br> $(8.5-12.4)\cdot E-3$ | $9.38\cdot E-5$ <br> $(7.86-12.0)\cdot E-5$ | 0.371 <br> (0.333−0.467) | 7.02 <br> (7.003−7.594) | 0.0468 <br> (0.0466−0.0486) |
| Mashonaland East | $1.123\cdot E-2$ <br> $(0.789-1.154)\cdot E-2$ | $6.853\cdot E-5$ <br> $(0.008-7.67)\cdot E-5$ | 0.263 <br> (0.176−0.61) | 7.219 <br> (7.001−7.228) | 0.105 <br> (0.104−0.109) |
| Midlands | $4.891\cdot E-3$ <br> $(4.575-5.633)\cdot E-3$ | $3.0\cdot E-5$ <br> $(2.57-4.04)\cdot E-5$ | 0.712 <br> (0.578−0.815) | 7.035 <br> (7.002−7.260) | 0.0689 <br> (0.0686−0.0703) |
| Manicaland | $4.734\cdot E-5$ <br> $(0.128-17.73)\cdot E-5$ | $1.401\cdot E-2$ <br> $(0.69-1.511)\cdot E-2$ | 0.976 <br> (0.852−0.999) | 8.881 <br> (7.19−13.72) | 0.0696 <br> (0.0685−0.0708) |
| Masvingo | $9.095\cdot E-3$ <br> $(7.265-9.140)\cdot E-3$ | $3.785\cdot E-6$ <br> $(0.078-8.585)\cdot E-6$ | 0.997 <br> (0.979−0.998) | 7.024 <br> (7.006−7.984) | 0.102 <br> (0.101−0.105) |
| Matabeleland North | $1.036\cdot E-5$ <br> $(0.361-52.0)\cdot E-5$ | $2.047\cdot E-3$ <br> $(0.70-2.15)\cdot E-3$ | 0.78 <br> (0.092−0.90) | 7.75 <br> (7.07−13.62) | 0.082 <br> (0.081−0.101) |
| Matabeleland South | $2.652\cdot E-3$ <br> $(2.642-4.257)\cdot E-3$ | $2.162\cdot E-2$ <br> $(2.0-3.2)\cdot E-2$ | 0.9797 <br> (0.87−0.998) | 7.3 <br> (7.018−8.998) | 0.0414 <br> (0.0387−0.043) |
