# Supplementary material for: An Optimal Cost Effectiveness Study on Zimbabwe Cholera Seasonal Data from 2008–2011
Source: PLoS One. 2013 Dec 3;8(12):e81231. doi: 10.1371/journal.pone.0081231 (PMC3849194; doi:10.1371/journal.pone.0081231)
Supplement: Table S4 — Estimated initial demographic variables of the cholera model (1). All data are given in the format [estimate (95% CI)]. (PDF) [file pone.0081231.s006.pdf]

Zimbabwe province	$S(0)(person)$	$I(0)(person)$	$R(0)(person)$	$B_H(0)\left(\frac{\text{cells}}{\text{mL}}\right)$	$B_L(0)\left(\frac{\text{cells}}{\text{mL}}\right)$	$C(0)(person)$	$D(0)(person)$
Harare	934947<br>(809355−1354411)	122<br>(4−192)	129<br>(7−147)	59000<br>(42184−79999)	2955<br>(54−8699)	106<br>(1−119)	7<br>(0−24)
Bulawayo	324497<br>(303446−674715)	8<br>(0−9)	3<br>(3−6)	417273<br>(38725−422806)	7945<br>(206−21243)	1<br>(0−9)	3<br>(0−3)
Mashonaland West	978852<br>(762909−1273118)	20<br>(1−39)	4<br>(0−15)	48641<br>(24945−90623)	13671<br>(171−25332)	26<br>(1−38)	0<br>(0−5)
Mashonaland Central	791525<br>(606360−846991)	4<br>(0−8)	5<br>(1−21)	2<br>(0−17)	20680383<br>(15675590−31190398)	1<br>(1−30)	1<br>(0−4)
Mashonaland East	712785<br>(706089−958344)	119<br>(115−125)	30<br>(2−78)	18<br>(17−77)	7147787<br>(1177−7755845)	60<br>(0−116)	11<br>(8−12)
Midlands	1397804<br>(1221465−1461080)	4<br>(0−7)	17<br>(3−19)	1<br>(0−11)	2726527<br>(2027236−2875339)	0<br>(0−32)	0<br>(0−3)
Manicaland	1070249<br>(1014932−1645816)	4<br>(0−5)	2<br>(0−3)	53965<br>(41117−82823)	1100<br>(89−12375)	1<br>(0−5)	1<br>(0−1)
Masvingo	710368<br>(708835−857436)	33<br>(30−39)	5<br>(0−20)	487<br>(478−658)	555732<br>(780−1016045)	10<br>(1−38)	1<br>(0−3)
Matabeleland North	512234<br>(301820−502397)	2<br>(0−2)	1<br>(0−2)	199051<br>(54911−318912)	803<br>(614−668969)	1<br>(0−2)	0<br>(0−1)
Matabeleland South	328003<br>(241114−328550)	370<br>(337−703)	444<br>(40−781)	5715<br>(3717−27562)	28187<br>(755−28641)	1252<br>(1157−1299)	50<br>(42−50)
