# Supplementary material for: An Optimal Cost Effectiveness Study on Zimbabwe Cholera Seasonal Data from 2008–2011
Source: PLoS One. 2013 Dec 3;8(12):e81231. doi: 10.1371/journal.pone.0081231 (PMC3849194; doi:10.1371/journal.pone.0081231)
Supplement: Appendix S1 — Details on the mathematical stability analysis of the cholera model (1). Details on the estimation procedure of the basic reproduction number () in periodic environment. Details on the intervention cost optimization procedure. (PDF) [file pone.0081231.s007.pdf]

Appendix S1

Formulation of the basic model without any control

Two routes of cholera transmission have been described in the literature, primary and secondary transmission. Primary transmission occurs due to an exposure to the contaminated environmental reservoir which contains *V. cholerae* bacteria. This scenario was mathematically modelled by Codeço *et.al.*[1]. Primary infection immensely depends upon the climatic and environmental factors that affect the seasonal pattern of infection [2,3,4,5,6,7,8]. In locations like Africa and South America, where, one yearly peak of cholera is often observed, the beginning of the epidemics has been associated with environmental conditions that favor the growth and survival of the bacterium [1,9]. Primary transmission plays only a limited role in an epidemiological process since it does not fully explain the exponential growth of incidences during epidemics.

Merrell *et.al.*[10] showed that freshly shed *V. cholerae* from human intestines out competes other *V. cholerae* by as much as 700-fold for at least the first 5 hours in the environment. This scenario is modelled by Hartley *et.al.*[11] by modifying the earlier model by Codeço *et.al.*[1] to explicitly account for the concentration of hyper-infectious bacteria within the drinking water. Associating higher levels of hyper-infectious concentrations with increased human-to-human transmission, the authors illustrate the importance of this additional transmission route (secondary transmission) in modelling the explosive epidemics, often associated with cholera [11].

We extend the model by Hartley *et.al.*[11], by assuming periodic contact rate in both transmission (primary and secondary), as seen in most of the cholera affected African and South American countries where one yearly peak of cholera is often observed. So we assume that the time period of transmission rate varies over 52-week period and also incorporates cholera-related death rate, which was also neglected. We consider the total human population size at time t is denoted by $N(t)$, which consists of susceptible human $S(t)$, infected human, $I(t)$ and recovered human, $R(t)$. Total bacteria population at time t consists of hyper-infectious bacteria $B_H(t)$ and low-infectious bacteria $B_L(t)$.

Susceptible population is increased by constant recruitment of newborn at a rate Π_H and by those recovered populations who loses their temporary immunity to cholera at a rate ω. Susceptible human is reduced by getting infected on contact with hyper-infectious and low-infectious bacterium at rates $\beta_H(t)\frac{B_H}{K_H+B_H}$ and $\beta_L(t)\frac{B_L}{K_L+B_L}$ and also decreased by natural death at a rate μ_d.

Infected human is increased by those susceptible humans who got infected in contact with hyper-infectious and low-infectious vibrios and decreased by those who recovered from cholera at rate γ, those who die due to cholera infection at a rate μ_c and those who die naturally at a rate μ_d.

Recovered human population is increased by those infected people who got recovery from the disease at a rate γ and reduced due to loss of natural immunity to cholera at a rate ω and who dies naturally at a rate μ_d.

Hyper-infectious bacterium is enriched from the amount of HI *V. cholerae* bacterium in the contaminated aquatic environment due to infected human feces at a rate ξ and diminish due to decay from hyper-infectious state to low-infectious state at a rate χ.

We assume that hyper-infectious bacterium losses their hyper infectivity at a rate χ to become low infectious and their natural death rate in aquatic environment is δ_L.

Based on the assumptions stated above, we can now write down the following system of nonlinear differential equations:

$$
\begin{aligned}
\frac{dS}{dt} &= \Pi_H + \omega R - \beta_H(t)\frac{B_H S}{K_H+B_H} - \beta_L(t)\frac{B_L S}{K_L+B_L} - \mu_d S \\
\frac{dI}{dt} &= \beta_H(t)\frac{B_H S}{K_H+B_H} + \beta_L(t)\frac{B_L S}{K_L+B_L} - (\gamma+\mu_d+\mu_c)I \\
\frac{dR}{dt} &= \gamma I - (\omega+\mu_d)R \\
\frac{dB_H}{dt} &= \xi I - \chi B_H \\
\frac{dB_L}{dt} &= \chi B_H - \delta_L B_L
\end{aligned}
\tag{S.1}
$$

where,

$$\begin{aligned}\beta_H(t) &= \beta_{H0}\{1+\tfrac{\delta}{2}(1+\cos(\tfrac{2\pi t}{52}))\}\\ \beta_L(t) &= \beta_{L0}\{1+\tfrac{\delta}{2}(1+\cos(\tfrac{2\pi t}{52}))\}\end{aligned} \tag{S.2}$$

Parameter values of the basic model (S.1)

For biological feasibility, we assumed all parameters to be non-negative. The description of the model (S.1) parameters and their interpretation are given in the **Table S1**. Estimated values of the model (S.1) parameters (β_{H0}, β_{L0}, δ, ξ and μ_c) for each province of Zimbabwe are given in **Table S3** in the format estimate (95% CI). **Table S4** contains estimated initial conditions of the model (S.1) in the same format as in **Table S3**.

Basic properties of the cholera model (S.1) without control

We claim the following result:

Theorem 1 *The solutions ($S(t)$, $I(t)$, $R(t)$, $B_H(t)$, $B_L(t)$) of the model (S.1) are uniformly and ultimately bounded in $\mathbb{R}^5_+$, i.e. there exist an $L > 0$ and a $T > 0$ such that ($S(t)$, $I(t)$, $R(t)$, $B_H(t)$, $B_L(t)$)$\leq$ (L, L, L, L, L) for all $t \geq T$.*

Proof: Adding first three equations we get

$$\begin{aligned}\tfrac{dN}{dt} &= \Pi_H - \mu_d N - \mu_c I.\\ &\leq \Pi_H - \mu_d N\end{aligned}$$

Hence, by standard comparison theorem [12], there exist $t_1 > 0$, such that $N(t) \leq \frac{\Pi_H}{\mu_d}$ for all $t \geq t_1$. Thus we have $S(t) \leq \frac{\Pi_H}{\mu_d}$, $I(t) \leq \frac{\Pi_H}{\mu_d}$ and $R(t) \leq \frac{\Pi_H}{\mu_d}$ for all $t \geq t_1$. Now by fourth equation we have,

$$\tfrac{dB_H}{dt} \leq \tfrac{\xi \Pi_H}{\mu_d} - \chi B_H.$$

So again by comparison theorem [12], there exist $t_2 \geq t_1$ such that $B_H(t) \leq \frac{\xi \Pi_H}{\chi \mu_d}$ for all $t \geq t_2$.
Similarly from the fifth equation, there exist $T \geq t_1, t_2$ such that $B_L(t) \leq \frac{\xi \Pi_H}{\delta_L \mu_d}$ for all $t \geq T$.
Let $L = \max\{\frac{\Pi_H}{\mu_d}, \frac{\xi \Pi_H}{\chi \mu_d}, \frac{\xi \Pi_H}{\delta_L \mu_d}\}$.

Thus it follows, $S(t) \leq L$, $I(t) \leq L$, $R(t) \leq L$, $B_H(t) \leq L$, $B_L(t) \leq L$ for all $t \geq T$.

Therefore the solution of the system (S.1) are uniformly and ultimately bounded in $\mathbb{R}^5_+$. □

Existence and local stability of the disease free periodic state

The Model (S.1) has a unique disease free equilibrium given by:

$$E_0 = (\tfrac{\Pi_H}{\mu_d}, 0, 0, 0, 0). \tag{S.3}$$

To prove local stability we first calculate the basic reproduction number (R_0) of the system (S.1) according to the procedure presented by Wang and Zhao (2008) [13].
Following [13], we calculate the matrix of new infection as:

$$F(t)=\begin{bmatrix} 0 & \frac{\beta_H(t)\Pi_H}{K_H\mu_d} & \frac{\beta_L(t)\Pi_H}{K_L\mu_d} \\ 0 & 0 & 0 \\ 0 & 0 & 0 \end{bmatrix}$$

and the transmission matrix as:

$$V(t)=\begin{bmatrix} (\gamma+\mu_c+\mu_d) & 0 & 0 \\ -\xi & \chi & 0 \\ 0 & -\chi & \delta_L \end{bmatrix}$$

Let, $Y(t,s)$, $t \geq s$ be the evolution operator of the linear ϖ-periodic system

$$\frac{dy}{dt} = -V(t)y \tag{S.4}$$

That is, for each $s \in \mathbb{R}$, the 3×3 matrix $Y(t,s)$ satisfies

$$\frac{d}{dt}Y(t,s) = -V(t)Y(t,s),$$

for all $t \geq s$ and $Y(s,s) = \mathbf{I}$, where $\mathbf{I}$ is the 3×3 identity matrix.

Let C_ϖ be the ordered Banach space of all ϖ-periodic functions from $\mathbb{R}$ to $\mathbb{R}^3$ which is equipped with maximum norm $\|.\|_\infty$ and the positive cone $C_\varpi^+ = \{\phi \in C_\varpi\colon \phi(t) \geq 0$, for all t in $\mathbb{R}\}$. Consider the following linear operator $L : C_\varpi \to C_\varpi$ by

$$(L\phi)(t) = \int_0^{+\infty} Y(t,t-a)F(t-a)\phi(t-a)\mathrm{d}a \tag{S.5}$$

Following Wang and Zhao (2008) [13], we call L the next infection operator, and define basic reproduction number as $R_0 := \rho(L)$, where $\rho(L)$ is the spectral radius of L.

Biologically this operator signifies that, if $\phi(s)$, ϖ-periodic in s, be the initial distribution of infectious individuals. Then $F(s)\phi(s)$ is the rate of new infections produced by the infected individuals who were introduced at time s. Given $t \geq s$, then $Y(t,s)F(s)\phi(s)$ gives the distribution of those infected individuals who were newly infected at time s and remain in the infected compartment at time t. It follows that, operator (S.5) implies the distribution of accumulative new infections at time t produced by all those infected individuals $\phi(s)$ introduced at some time previous to t.

Motivated by the concept of partial reproduction number defined by Mukandavire *et.al.*[14], we also introduced two partial reproductive numbers for our system as: $R_h := \rho(L_1)$ and $R_l := \rho(L_2)$, where operator L_1 and L_2 are given as follows:

$$(L_1\phi_1)(t) = \int_0^{+\infty} Y(t,t-a)F_1(t-a)\phi_1(t-a)\mathrm{d}a \tag{S.6}$$

and

$$(L_2\phi_2)(t) = \int_0^{+\infty} Y(t,t-a)F_2(t-a)\phi_2(t-a)\mathrm{d}a \tag{S.7}$$

where, incidence matrices, F_1 and F_2 are given as follows:

$$F_1(t) = \begin{bmatrix} 0 & \frac{\beta_H(t)\Pi_H}{K_H\mu_d} & 0 \\ 0 & 0 & 0 \\ 0 & 0 & 0 \end{bmatrix}$$

and

$$F_2(t) = \begin{bmatrix} 0 & 0 & \frac{\beta_L(t)\Pi_H}{K_L\mu_d} \\ 0 & 0 & 0 \\ 0 & 0 & 0 \end{bmatrix}$$

Numerically, R_0, R_h and R_l for our periodic system (S.1) can be calculated according to following procedure: let $W(t,\lambda)$ be the fundamental matrix of the linear ϖ-periodic system

$$\frac{dw}{dt} = (-V(t) + \frac{F(t)}{\lambda})w, \tag{S.8}$$

$t \in \mathbb{R}$ with parameter $\lambda \in (0,\infty)$.

Since $F(t)$ is nonnegative and $-V(t)$ is cooperative (off diagonal element are nonnegative), it follows that $\rho(W(\varpi,\lambda))$ is continuous and non-increasing in $\lambda \in (0,\infty)$, and $\lim_{\lambda\to\infty}\rho(W(\varpi,\lambda)) < 1$. It is easy to verify that system (S.1) satisfies assumptions (A1)-(A7) in [13]. Thus we have the following two results

Lemma 1 *(Wang and Zhao (2008)[13], Theorem 2.1) The following statements are valid:*

(i) If $\rho(W(\varpi,\lambda)) = 1$ has a positive solution λ_0, then λ_0 is an eigenvalue of operator L, defined in (S.5), and hence $R_0 > 0$.
(ii) If $R_0 > 0$, then $\lambda = R_0$ is the unique solution of $\rho(W(\varpi,\lambda)) = 1$.
(iii) If $R_0 = 0$ if and only if $\rho(W(\varpi,\lambda)) < 1$ for all $\lambda > 0$.

Lemma 2 *(Wang and Zhao (2008)[13], Theorem 2.2) The following statements are valid:*

(i) $R_0 = 1$ if and only if $\rho(\Phi_{F-V}(\varpi)) = 1$.
(ii)$R_0 > 1$ if and only if $\rho(\Phi_{F-V}(\varpi)) > 1$.
(iii)$R_0 < 1$ if and only if $\rho(\Phi_{F-V}(\varpi)) < 1$.

Lemma 1 is used to calculate R_0, R_h, R_l numerically and using Lemma 2 we have the following result:

Theorem 2 *If $R_0 < 1$ disease free equilibrium (E_0) of system (S.1) is locally asymptotically stable and if $R_0 > 1$, then it is unstable.*

Global stability of the disease free state

Let $(\mathbb{R}^k, \mathbb{R}^k_+)$ be the standard ordered k-dimensional euclidian space with norm $\|.\|$. For $u, v \in \mathbb{R}^k$, we write $u \geq v$ provided $u - v \in \mathbb{R}^k_+$, $u > v$ provided $u - v \in \mathbb{R}^k_+ \setminus \{0\}$, and $u \gg v$ provided $u - v \in \text{Int}(\mathbb{R}^k_+)$.

Recall that a $k \times k$ matrix $A = (a_{ij})$ is said to be irreducible if its index set $\{1, 2, ..., k\}$ cannot be split into two complementary sets (without common indices) $\{m_1, m_2, ..., m_{\nu_1}\}$ and $\{n_1, n_2, ..., n_{\nu_2}\}$ $(\nu_1 + \nu_2 = k)$ such that $a_{m_p n_q} = 0 \ \forall\ 1 \leq p \leq \nu_1,\ 1 \leq q \leq \nu_2$.

Let $A(t)$ be continuous, cooperative and irreducible and ϖ-periodic $k \times k$ matrix function, $\Phi_{A(.)}(t)$ be the fundamental matrix solution of the linear ordinary differential equation

$$\frac{dx}{dt} = A(t)x \tag{S.9}$$

and $\rho(\Phi_{A(.)}(\varpi))$ be the spectral radius of the monodromy matrix $\Phi_{A(.)}(\varpi)$. It then follows from [15,16], that $\Phi_{A(.)}(t)$ is a matrix with all entries positive for each $t > 0$. Therefore, by Perron-Frobenius theorem, $\rho(\Phi_{A(.)}(\varpi))$ is the principal eigenvalue of $\Phi_{A(.)}(\varpi)$ in the sense that it is simple and admits an eigenvector $v^* \gg 0$. The following result by Zhang and Zhao (2007)[17], is useful to prove global stability of E_0 and in upcoming sections.

Lemma 3 *(Zhang and Zhao (2007)[17], Lemma 2.1) Let* $s = \frac{1}{\varpi} \ln \rho(\Phi_{A(.)}(\varpi))$. *Then there exists a positive, ϖ-periodic function $f(t)$ such that $e^{st} f(t)$ is a solution of (S.9).*

We claim the following:

Theorem 3 *If $R_0 < 1$ then disease free periodic state E_0 is globally asymptotically stable.*

Proof: From Theorem 2 we have if $R_0 < 1$, disease free equilibrium E_0 of the system (S.1) is locally asymptotically stable, therefore it is sufficient to show that E_0 is globally asymptotically stable for $R_0 < 1$.

From second, forth and last equations of (S.1) we have,

$$\begin{aligned}
\frac{dI}{dt} &= \beta_H(t)\frac{B_H S}{K_H + B_H} + \beta_L(t)\frac{B_L S}{K_L + B_L} - (\gamma + \mu_d + \mu_c)I \\
&\leq \beta_H(t)\frac{\Pi_H}{\mu_d}\frac{B_H}{K_H + B_H} + \beta_L(t)\frac{\Pi_H}{\mu_d}\frac{B_L}{K_L + B_L} - (\gamma + \mu_d + \mu_c)I \\
&\leq \beta_H(t)\frac{\Pi_H}{\mu_d K_H}B_H + \beta_L(t)\frac{\Pi_H}{\mu_d K_L}B_L - (\gamma + \mu_d + \mu_c)I \\
\frac{dB_H}{dt} &= \xi I - \chi B_H \\
\frac{dB_L}{dt} &= \chi B_H - \delta_L B_L,
\end{aligned} \tag{S.10}$$

$\forall\ t \geq 0$, as $0 \leq S(t) \leq \frac{\Pi_H}{\mu_d}$ and $B_H \geq 0$.

Now, consider the auxiliary system:

$$\begin{aligned}
\frac{dI}{dt} &= \beta_H(t)\frac{\Pi_H}{\mu_d K_H}B_H + \beta_L(t)\frac{\Pi_H}{\mu_d K_L}B_L - (\gamma + \mu_d + \mu_c)I \\
\frac{dB_H}{dt} &= \xi I - \chi B_H \\
\frac{dB_L}{dt} &= \chi B_H - \delta_L B_L
\end{aligned} \tag{S.11}$$

Which can be written as:

$$\frac{dX}{dt} = (F(t) - V(t))X, \tag{S.12}$$

where $X = (I(t), B_H(t), B_L(t))^T$.

Now $(F(t) - V(t))$ is continuous, cooperative and irreducible, therefore by Lemma 3, there exists a positive, ϖ-periodic function $\bar{X}(t)$ such that $X(t) = e^{st}\,\bar{X}(t)$, is a solution of the system (S.12), where $s = \frac{1}{\varpi} \ln \rho(\Phi_{F(.)-V(.)}(\varpi))$. From Lemma 2, if $R_0 < 1$, $\rho(\Phi_{F(.)-V(.)}(\varpi)) < 1$, thus s is a negative constant. Therefore, we have $X(t) \to 0$ as $t \to +\infty$.

Thus zero solution of (S.12) is globally asymptotically stable. For any non-negative initial value $(I(0), B_H(0), B_L(0))^T$ of the system (S.10), there is a sufficiently large $M > 0$ such that $(I(0), B_H(0), B_L(0))^T \leq M\bar{X}(0)$ holds. Applying the comparison principle [18], we have $(I(t), B_H(t), B_L(t))^T \leq M\bar{X}(t)$, for all $t > 0$, where $M\bar{X}(t)$ is also a solution of (S.12). Therefore, we get $I(t) \to 0$, $B_H(t) \to 0$ and $B_L(t) \to 0$ as $t \to \infty$. Using theory of asymptotic autonomous systems [19], it follows then $S(t) \to \frac{\Pi_H}{\mu_d}$ and $R(t) \to 0$ as $t \to \infty$.

Thus E_0 is globally asymptotically stable if $R_0 < 1$. □

Uniform persistence of the disease

We claim the following

Theorem 4 *If $R_0 > 1$, the system (S.1) is uniformly persistent, i.e., there exists a positive constant ϵ, such that for all initial value $(S(0), I(0), R(0), B_H(0), B_L(0)) \in \{(S, I, R, B_H, B_L) \in \mathbb{R}^5_+ : I > 0, B_H > 0, B_L > 0\}$, the solution of (S.1) satisfies $\liminf_{t\to\infty} I(t) \geq \epsilon$, $\liminf_{t\to\infty} B_H(t) \geq \epsilon$ and $\liminf_{t\to\infty} B_L(t) \geq \epsilon$.*

Proof: Consider the sets in $\mathbb{R}^5$, $X = \mathbb{R}^5_+$, $X_0 = \{(S, I, R, B_H, B_L) \in \mathbb{R}^5_+ : I > 0, B_H > 0, B_L > 0\}$ and $\partial X_0 = X \backslash X_0$.

We define a poincarè map $P : X \to X$, satisfying $P(x^0) = u(\varpi, x^0)$, $\forall\, x^0 \in X$, with $u(t, x^0)$ the unique solution of (S.1) satisfying $u(0, x^0) = x^0$.

We first show that P is uniformly persistent with respect to $(X_0, \partial X_0)$. It is easy to see from the system (S.1) that X and X_0 are positively invariant. Moreover, ∂X_0 is relatively closed set in X. It then follows from Theorem 1 that solutions of the system (S.1) are uniformly and ultimately bounded. Therefore, the semiflow P is point dissipative and compact on X. It follows from Theorem 1.1.3 in [20], there is a global attractor of P that attracts each bounded set in X.

We define the following set:

$$M_\partial = \{(S(0), I(0), R(0), B_H(0), B_L(0)) \in \partial X_0 :$$
$$P^m(S(0), I(0), R(0), B_H(0), B_L(0)) \in \partial X_0, \forall m \in \mathbb{N} \cup \{0\}\}$$

We claim that the following

$$M_\partial = \{(S, 0, R, 0, 0) : S \geq 0, R \geq 0\}$$

We only need to show that, $M_\partial \subseteq \{(S, 0, R, 0, 0) : S \geq 0, R \geq 0\}$.

For any, $(S(0), I(0), R(0), B_H(0), B_L(0)) \in \partial X_0 \backslash \{(S, 0, R, 0, 0) : S \geq 0, R \geq 0\}$, if $I(0) = 0$, $B_H = 0$, $B_L > 0$, it is clear that $S > 0$ and $B_L > 0$ for all $t > 0$, now from second equation of (S.1), we have $\dot{I}(0) = \beta_H(t)\frac{S(0)B_L(0)}{K_L + B_L(0)} > 0$ $\Rightarrow I(0) > 0$. Thus from fourth equation of (S.1), we have $\dot{B_H}(0) = \xi I(0) > 0$. else if $I(0) = 0$, $B_L = 0$ and $B_H > 0$ then similarly we can show $\dot{I}(0) > 0$ and $\dot{B_H}(0) > 0$ and similarly for other cases also. Therefore if $(S(0), I(0), R(0), B_H(0), B_L(0)) \notin \{(S, 0, R, 0, 0) : S \geq 0, R \geq 0\}$ then $(S(t), I(t), R(t), B_H(t), B_L(t)) \notin \partial X_0$ for sufficiently small $t > 0$ *i.e.* for any $(S(0), I(0), R(0), B_H(0), B_L(0)) \notin \{(S, 0, R, 0, 0) : S \geq 0, R \geq 0\}$ then $(S(0), I(0), R(0), B_H(0), B_L(0)) \notin \partial X_0$. This implies $M_\partial \subseteq \{(S, 0, R, 0, 0) : S \geq 0, R \geq 0\}$ and therefore $M_\partial = \{(S, 0, R, 0, 0) : S \geq 0, R \geq 0\}$

Clearly, E_0 is a fixed point of P in M_∂. If $(S(t), I(t), R(t), B_H(t), B_L(t))$ is a solution of (S.1) initiating from M_∂, it then follows from system (S.1) that $S(t) \to \frac{\Pi_H}{\mu_d}$, $I(t) \to 0$, $R(t) \to 0$, $B_H(t) \to 0$ and $B_L(t) \to 0$ as $t \to \infty$. So any solution of (S.1) initiating in M_∂ will remain into M_∂.

We will now show that $\{E_0\}$ is an acyclic covering of E_0. It is enough to show $\{E_0\}$ isolated invariant subset of M_∂ *i.e.* $W^s(E_0) \bigcap X_0 = \emptyset$, where $W^s(E_0)$ is the stable set of E_0.

Let $x^0 = (S(0), I(0), R(0), B_H(0), B_L(0)) \in X_0$, then by the continuity of solution with respect to initial values, $\forall\, \varepsilon \in (0, \frac{\Pi_H}{\mu_d})$, there exits $\eta > 0$ such that $\forall\, x^0 \in X_0$ with $\|x^0 - E_0\| \leq \eta$, it follows that $\|u(t, x^0) - u(t, E_0)\| \leq \varepsilon$, $\forall\, t \in [0, \varpi]$. To show, $x^0 \in X_0 \Rightarrow x^0 \notin W^s(E_0)$, it is enough to show that $\limsup_{m\to\infty} d(P^m(x^0), E_0) \geq \eta$ for some $m > 0$. If not, let $\exists x^0 \in X_0$ such that $\limsup_{m\to\infty} d(P^m(x^0, E_0) < \eta$ for all $m > 0$. This implies $\|u(t, P^m(x^0)) - u(t, E_0)\| < \varepsilon$, $\forall\, t \in [0, \varpi]$.

For any $t \geq 0$, let $t = m\varpi + t_1$, where $t_1 \in [0, \varpi]$ and $m = [\frac{t}{\varpi}]$, which is the greatest integer less than or equal to $\frac{t}{\varpi}$. Therefore, we have, $\|u(t, P^m(x^0)) - u(t, E_0)\| = \|u(t_1, P^m(x^0)) - u(t_1, E_0)\| < \varepsilon, \forall\, t \in [0, \varpi]$.

Replacing, $u(t, x^0)$ by $(S(t), I(t), R(t), B_H(t), B_L(t))$ in above equation, it follows that, $\frac{\Pi_H}{\mu_d} - \varepsilon \leq S(t) \leq \frac{\Pi_H}{\mu_d} + \varepsilon$, $0 \leq I(t) \leq \varepsilon$, $0 \leq R(t) \leq \varepsilon$, $0 \leq B_H(t) \leq \varepsilon$ and $0 \leq B_L(t) \leq \varepsilon$ for all $t \geq 0$. Then we have, $\frac{S(t)}{K_H + B_H} \geq (\frac{\Pi_H}{\mu_d K_H} - \frac{\varepsilon}{K_H + \varepsilon})$ and $\frac{S(t)}{K_L + B_L} \geq (\frac{\Pi_H}{\mu_d K_L} - \frac{\varepsilon}{K_L + \varepsilon})$. Therefore, from system (S.1), we have

$$\begin{aligned} \frac{dI}{dt} &\geq \beta_H(t)(\frac{\Pi_H}{\mu_d K_H} - \frac{\varepsilon}{K_H + \varepsilon})B_H + \beta_L(t)(\frac{\Pi_H}{\mu_d K_L} - \frac{\varepsilon}{K_L + \varepsilon})B_L \\ &- (\gamma + \mu_d + \mu_c)I \\ \frac{dB_H}{dt} &= \xi I - \chi B_H \\ \frac{dB_L}{dt} &= \chi B_H - \delta_L B_L \end{aligned} \tag{S.13}$$

Set

$$M_\varepsilon(t) = \begin{bmatrix} 0 & \beta_H(t)\frac{\varepsilon}{K_H + \varepsilon} & \beta_L(t)\frac{\varepsilon}{K_L + \varepsilon} \\ 0 & 0 & 0 \\ 0 & 0 & 0 \end{bmatrix}.$$

Again by Lemma 2, we have as $R_0 > 1$ so $\rho(\Phi_{F(.)-V(.)}(\varpi)) > 1$, choosing ε sufficiently small such that $\rho(\Phi_{F(.)-V(.)-M_\varepsilon}(\varpi)) > 1$. Again by Lemma 3 and comparison principle [18], there exists a positive ϖ-periodic function $f_1(t)$ such that $x(t) \geq f_1(t)e^{s_2 t}$, where $x(t) = (I(t), B_H(t), B_L(t))^T$ and $s_2 = \frac{1}{\varpi}\ln \rho(\Phi_{F(.)-V(.)-M_\varepsilon}(\varpi)) > 0$, which implies that $I(t) \to \infty$, $B_H(t) \to \infty$ and $B_L(t) \to \infty$, as $t \to \infty$, this is contradiction in M_∂, hence $W^s(E_0) \bigcap X_0 = \emptyset$ and therefore $\{E_0\}$ is an acyclic covering of E_0 in M_∂. Therefore from Theorem 1.3.1 and Remark 1.3.1 in [20], we obtain that P is uniformly persistent with respect to $(X_0, \partial X_0)$. Therefore it follows from Theorem 3.1.1 in [20], that the solution of the system (S.1) is uniformly persistent in X if $R_0 > 1$. □

Existence and global stability of periodic solution of the cholera model (S.1)

We claim the following:

Theorem 5 *If $R_0 > 1$, then the system (S.1) have a positive ϖ-periodic solution which is globally asymptotically stable.*

Proof: We have already proved in last section that the poincaѐ map, $P : X \to X$ of system (S.1) is point dissipative and compact and by Theorem 4, P is uniformly persistent with respect to $(X_0, \partial X_0)$. Then it follows from Theorem 1.3.6 in [20], that the poincaѐ map P has a fixed point $(\bar{S}, \bar{I}, \bar{R}, \bar{B}_H, \bar{B}_L) \in Int(\mathbb{R}^5_+)$. Hence $u(t, (\bar{S}, \bar{I}, \bar{R}, \bar{B}_H, \bar{B}_L)) \in Int(\mathbb{R}^5_+)$, $\forall\, t > 0$. Thus $(\bar{S}(t), \bar{I}(t), \bar{R}(t), \bar{B}_H(t), \bar{B}_L(t))$ is a positive ϖ-periodic solution of the system (S.1) by the definition of the semiflow P.

Now to prove global stability of the periodic solution $(\bar{S}(t), \bar{I}(t), \bar{R}(t), \bar{B}_H(t), \bar{B}_L(t))$ of the model (S.1) we construct the following Lyapunov function $L(t) = |S(t) - \bar{S}(t)| + |I(t) - \bar{I}(t)| + |R(t) - \bar{R}(t)| + \frac{\mu_d}{\xi}|B_H(t) - \bar{B}_H(t)| + \frac{\mu_d}{\xi}|B_L(t) - \bar{B}_L(t)|$. We use the formula,

$$|x|' = \text{sgn}(x)\; x'$$

to calculate the upper right-hand derivative (Dini's Derivative) of $L(t)$.

Therefore,

$$
\begin{aligned}
D^+L(t) &= \mathrm{sgn}(S(t)-\bar{S}(t))\{\beta_H(t)\tfrac{\bar{B}_H\bar{S}}{K_H+\bar{B}_H} \\
&+ \beta_L(t)\tfrac{\bar{B}_L\bar{S}}{K_L+\bar{B}_L} - \beta_H(t)\tfrac{B_H S}{K_H+B_H} \\
&- \beta_L(t)\tfrac{B_L S}{K_L+B_L} + \omega(R-\bar{R}) - \mu_d(S-\bar{S})\} \\
&+ \mathrm{sgn}(I(t)-\bar{I}(t))\{\beta_H(t)\tfrac{B_H S}{K_H+B_H} + \beta_L(t)\tfrac{B_L S}{K_L+B_L} \\
&- \beta_H(t)\tfrac{\bar{B}_H\bar{S}}{K_H+\bar{B}_H} - \beta_L(t)\tfrac{\bar{B}_L\bar{S}}{K_L+\bar{B}_L} - (\gamma+\mu_c+\mu_d)(I-\bar{I})\} \\
&+ \mathrm{sgn}(R(t)-\bar{R}(t))\{\gamma(I-\bar{I}) - (\omega+\mu_d)(R-\bar{R})\} \\
&+ \mathrm{sgn}(B_H(t)-\bar{B}_H(t))\tfrac{\mu_d}{\xi}\{\xi(I-\bar{I}) - \chi(B_H-\bar{B}_H)\} \\
&+ \mathrm{sgn}(B_L(t)-\bar{B}_L(t))\tfrac{\mu_d}{\xi}\{\chi(B_H-\bar{B}_H) - \delta_L(B_L-\bar{B}_L)\} \\
&\le -\mu_d|S(t)-\bar{S}(t)| - \mu_c|I(t)-\bar{I}(t)| - \mu_d|R(t)-\bar{R}(t)| \\
&- \tfrac{\mu_d\delta_L}{\xi}|B_L(t)-\bar{B}_L(t)|
\end{aligned}
\tag{S.14}
$$

Let $K=\min\{\mu_d,\mu_c,\delta_L\}$, then it follows that

$$
\begin{aligned}
D^+L(t) \le -K(&|S(t)-\bar{S}(t)| + |I(t)-\bar{I}(t)| + |R(t)-\bar{R}(t)| \\
&+ |B_L(t)-\bar{B}_L(t)|)
\end{aligned}
$$

Which implies that L is non-increasing on $[0,+\infty)$. Integrating the above inequality from 0 to t we have,

$$
\begin{aligned}
&L(t) + K\int_0^t(|S(s)-\bar{S}(s)| + |I(s)-\bar{I}(s)| + |R(s)-\bar{R}(s)| \\
&+|B_L(s)-\bar{B}_L(s)|)\mathrm{d}s \le L(0) < +\infty,\ \forall t\ge 0
\end{aligned}
\tag{S.15}
$$

Thus following Lemma 2.2 in [21], we have, $\lim_{t\to\infty} L(t)=0$. Therefore it follows that

$\lim_{t\to\infty}|S(t)-\bar{S}(t)|=0$; $\lim_{t\to\infty}|I(t)-\bar{I}(t)|=0$; $\lim_{t\to\infty}|R(t)-\bar{R}(t)|=0$; $\lim_{t\to\infty}|B_H(t)-\bar{B}_H(t)|=0$; $\lim_{t\to\infty}|B_L(t)-\bar{B}_L(t)|=0$.

Thus, $(\bar{S}(t),\bar{I}(t),\bar{R}(t),\bar{B}_H(t),\bar{B}_L(t))$ is globally attracting.

Now we show that there exists only one ϖ- periodic solution of the system (S.1). For any two ϖ- periodic solutions $(\bar{S},\bar{I},\bar{R},\bar{B}_H,\bar{B}_L)$ and $(\bar{S}_1,\bar{I}_1,\bar{R}_1,\bar{B}_{H1},\bar{B}_{L1})$ of the system (S.1), we claim that $\bar{S}(t)=\bar{S}_1(t)$; $\bar{I}(t)=\bar{I}_1(t)$; $\bar{R}(t)=\bar{R}_1(t)$; $\bar{B}_H(t)=\bar{B}_{H1}(t)$ and $\bar{B}_H(t)=\bar{B}_{H1}(t)$, for all $t\in[0,\varpi]$. If not, then there must be at least one $\eta\in[0,\varpi]$ such that $\bar{S}(\eta)\neq\bar{S}_1(\eta)$ *i.e.* $|\bar{S}(\eta)-\bar{S}_1(\eta)|=\varepsilon>0$.

Thus we can get

$$
\begin{aligned}
\varepsilon &= \lim_{n\to\infty}|\bar{S}(\eta+n\varpi)-\bar{S}_1(\eta+n\varpi)| \\
&= \lim_{t\to\infty}|\bar{S}(t)-\bar{S}_1(t)| > 0
\end{aligned}
$$

Which is a contradiction to the fact that $(\bar{S},\bar{I},\bar{R},\bar{B}_H,\bar{B}_L)$ is globally stable. Therefore $\bar{S}(t)=\bar{S}_1(t)$, $\forall t\in[0,\varpi]$ and similarly for other cases also. Therefore, $\bar{S}(t)=\bar{S}_1(t)$; $\bar{I}(t)=\bar{I}_1(t)$; $\bar{R}(t)=\bar{R}_1(t)$; $\bar{B}_H(t)=\bar{B}_{H1}(t)$ and $\bar{B}_H(t)=\bar{B}_{H1}(t)$, for all $t\in[0,\varpi]$.

So if $R_0>1$, $\exists$ unique ϖ-periodic solution of the system (S.1) which is globally asymptotically stable. □

Cholera model with different interventions strategies

We consider four different interventions in the model (S.1) to reduce cholera burden namely, (i) Hand-hygiene promotion & clean water supply, (ii) Vaccination, (iii) Treatment using Antibiotic and oral rehydration therapy, and (iv) Construction and promotion of Sanitation.

Following the assumptions given in the section **Model with different cholera interventions** in the **main text**, we have the following cholera model with different interventions:

$$\begin{aligned}
\frac{dS}{dt} &= \Pi_H + \omega R + \epsilon V - (1-\theta(t))[\beta_H(t)\frac{B_H S}{K_H+B_H} + \beta_L(t)\frac{B_L S}{K_L+B_L}] \\
&- p(t)\sigma S - \mu_d S \\
\frac{dI}{dt} &= (1-\theta(t))[\beta_H(t)\frac{B_H S}{K_H+B_H} + \beta_L(t)\frac{B_L S}{K_L+B_L}] \\
&- (\mu_d + \mu_c + (1-\alpha(t))\gamma + \alpha(t)\gamma\lambda)I \\
\frac{dV}{dt} &= p(t)\sigma S - \epsilon V - \mu_d V \\
\frac{dR}{dt} &= ((1-\alpha(t))\gamma + \alpha(t)\gamma\lambda)I - (\omega+\mu_d)R \\
\frac{dB_H}{dt} &= (1-s(t))(\psi\alpha(t) + (1-\alpha(t)))\xi I - \chi B_H \\
\frac{dB_L}{dt} &= \chi B_H - \delta_L B_L
\end{aligned} \tag{S.16}$$

The description of the interventions parameters of the model (S.16) and their interpretations are given in the **Table S2**.

Optimal control strategy

In order to minimize the control objective function $J(\theta, p, \alpha, s)$ (see **main text Equation 15**), we apply Pontryagin's Maximum Principle [22] that allow us to study our state system S.16 to our control objective function. Fleming and Rishel (1975)[23], first showed that this principle can be used to obtain the differential equations for the adjoint variables, corresponding boundary conditions and the characterization of an optimal control estimates $\theta^*(t)$, $p^*(t)$, $\alpha^*(t)$ & $s^*(t)$. This characterization gives a representation of an optimal control in terms of the state and adjoint functions. Also, this principle converts the problem of minimizing the objective functional subject to state system into minimizing the Hamiltonian with respect to the controls (bounded measurable function) at each time t.

Forming the Hamiltonian, H, we have

$$\begin{aligned}
H &= A\mu_c I(t) + Bp(t)S(t) + Cp^2\sigma + D\alpha(t)I(t) + E\alpha^2(t) + F\theta(t) \\
&+ G\theta^2(t) + Hs(t) + Ks^2(t) + \lambda_S[\Pi_H + \omega R(t) \\
&- (1-\theta(t))\{\beta_H(t)\frac{B_H(t)S(t)}{K_H+B_H(t)} + \beta_L(t)\frac{B_L(t)S(t)}{K_L+B_L(t)}\} + \epsilon V(t) \\
&- p(t)\sigma S(t) - \mu_d S(t)] + \lambda_I[(1-\theta(t))\{\beta_H(t)\frac{B_H(t)S(t)}{K_H+B_H(t)} \\
&+ \beta_L(t)\frac{B_L(t)S(t)}{K_L+B_L(t)}\} - \{\mu_d + \mu_c + (1-\alpha(t))\gamma + \alpha(t)\gamma\lambda\}I(t)] \\
&+ \lambda_V[p(t)\sigma S(t) - \epsilon V(t) - \mu_d V(t)] \\
&+ \lambda_R[\{(1-\alpha(t))\gamma + \alpha(t)\gamma\lambda\}I(t) - (\omega+\mu_d)R(t)] \\
&+ \lambda_{BH}[(1-s(t))\{\psi\alpha(t) + (1-\alpha(t))\}\xi I(t) - \chi B_H(t)] \\
&+ \lambda_{BL}[\chi B_H(t) - \delta_L B_L(t)]
\end{aligned} \tag{S.17}$$

where λ_S, λ_I, λ_V, λ_R, λ_{BH} and λ_{BL} are adjoint functions associated with their respective states. First term in the Hamiltonian H comes from the integrand of the control objective functional (see **main text Equation 15**) and remaining terms are product of each adjoint function with the right-hand side of the differential equation of its corresponding state function.

Given an optimal control estimates $(\theta^*(t), p^*(t), \alpha^*(t), s^*(t))$ and corresponding states $(S^*, I^*, V^*, R^*, B_H^*, B_L^*)$, there exist adjoint functions satisfying following system of equations:

$$\begin{aligned}
\frac{d\lambda_S}{dt} &= p(t)(\sigma\lambda_S - \sigma\lambda_V - B) + (1-\theta(t))[\beta_H(t)\frac{B_H}{K_H+B_H} \\
&+ \beta_L(t)\frac{B_L}{K_L+B_L}](\lambda_S - \lambda_I) + \lambda_S\mu_d \\
\frac{d\lambda_I}{dt} &= (\lambda_I - A)\mu_c + (\lambda_I - \lambda_R)\{(1-\alpha(t))\gamma + \alpha(t)\gamma\lambda\} \\
&+ \lambda_I\mu_d - D\alpha(t) - \lambda_{BH}(1-s(t))\xi\{\psi\alpha(t) + (1-\alpha(t))\} \\
\frac{d\lambda_V}{dt} &= \epsilon(\lambda_V - \lambda_S) + \lambda_V\mu_d \\
\frac{d\lambda_R}{dt} &= \omega(\lambda_R - \lambda_S) + \lambda_R\mu_d \\
\frac{d\lambda_{BH}}{dt} &= (\lambda_S - \lambda_I)(1-\theta(t))\frac{\beta_H(t)K_H S}{(K_H+B_H)^2} + \chi(\lambda_{BH} - \lambda_{BL}) \\
\frac{d\lambda_{BL}}{dt} &= (\lambda_S - \lambda_I)(1-\theta(t))\frac{\beta_L(t)K_L S}{(K_L+B_L)^2} + \delta_L\lambda_{BL}
\end{aligned} \tag{S.18}$$

with final time boundary conditions

$$\lambda_S(T) = 0,\ \lambda_I(T) = 0,\ \lambda_V(T) = 0,\ \lambda_R(T) = 0,\ \lambda_{BH}(T) = 0,\ \lambda_{BL}(T) = 0.$$

Note that, $\frac{d\lambda_S}{dt} = -\frac{\partial H}{\partial S}$ and similarly for the other adjoint differential equations. The final time boundary conditions are zero since there is no dependence on the states at the final time in the objective functional.

Optimal control estimates are characterized as follows:

$$\theta^*(t) = \max(0, \min(\hat{\theta}(t), \theta_{\max}))$$

$$p^*(t) = \max(0, \min(\hat{p}(t), p_{\max}))$$

$$\alpha^*(t) = \max(0, \min(\hat{\alpha}(t), \alpha_{\max}))$$

and

$$s^*(t) = \max(0, \min(\hat{s}(t), s_{\max}))$$

where values of $\theta_{\max}$, $p_{\max}$, $\alpha_{\max}$ and $s_{\max}$ are given in **Table S2**. Expression of $\hat{\theta}(t)$, $\hat{p}(t)$, $\hat{\alpha}(t)$ and $\hat{s}(t)$ given as follows:

$$\begin{aligned}
\hat{\theta}(t) &= \frac{F + (\lambda_S - \lambda_I)\{\frac{\beta_H(t)B_H S}{K_H+B_H} + \frac{\beta_L(t)B_L S}{K_L+B_L}\}}{(-2G)}. \\
\hat{p}(t) &= \frac{(\lambda_V - \lambda_S + B)S(t)}{(-2C)}. \\
\hat{\alpha}(t) &= \frac{[\lambda_R\gamma(\lambda-1) + D - \lambda_I\gamma(\lambda-1) - \lambda_{BH}(1-s(t))\xi(1-\psi)]I(t)}{(-2E)}. \\
\hat{s}(t) &= \frac{H - \lambda_{BH}\{\psi\alpha(t) + (1-\alpha(t))\}\xi I(t)}{(-2K)}.
\end{aligned} \tag{S.19}$$

Note that $\hat{\theta}(t)$ comes from $\frac{\partial H}{\partial \theta} = 0$, similarly for other controls.

The state system of differential equations (S.16) and the adjoint system of differential equations (S.18) together with the control characterizations (S.19) is solved numerically in MATLAB 7.11.0 (R2010b) using the method of

steepest decent [24].

The optimal cost-effective study in Zimbabwe, from the period end of epidemic in *2008-09* to *January 1, 2012,* (see, **Tables 8** and **9** in **main text**) suggests that the vaccination coverage to control cholera epidemics may be a suitable option provided it is applied with the combinations of other interventions. This result agrees with the point made in a recent analysis [25] to control cholera epidemic in Haiti using vaccination. Higher values of the cost per case reduction (see, **Table 9** in **main text**) is needed if vaccination is applied alone, made the vaccination strategy as an impractical control strategy.

In terms of case reduction among single interventions, hand-hygiene & clean water distribution is the most effective intervention. It reduces the most number of cases and deaths among single interventions in each province in Zimbabwe during the projected intervention period *i.e.* the end of epidemic in *2008-09* to *January 1, 2012,* (see, **Tables 5** and **6** in **main text**). This result is in good agreement with the observation of Andrews and Basu [26], where they argued that hand-hygiene & clean water distributions will avert more cases and deaths than treatment and vaccination during the epidemic in Haiti. Hand-hygiene & clean water distribution is found to be the most cost effective among single interventions in those provinces (Harare, Mashonaland West, Manicaland, Matabeleland South and Matabeleland North) where slow transmission is a dominating factor for cholera transmission (see **Table 9** in **main text** and **Table S3**). Thus, the region where environmental factors play the key role in cholera transmission, hand-hygiene & clean water distribution may be the most cost effective intervention there.

Treatment as an individual control has turned out to be the most cost effective among different single interventions in the provinces Mashonaland Central, Mashonaland East, Midlands and Masvingo (see **Tables 8** in **main text**). This result is in well agreement with the previous observation of Naficy *et. al.* [27] on the control of cholera in sub-Saharan refugee settings. The provinces where treatment is found to be most cost effective one under the dominance of hyper-infectious cholera transmission (see **Table S3**). This result can be very helpful for policy makers to choose a particular intervention during a cholera epidemic in a particular region.

Sanitation as a single intervention performed better than vaccination. However, in terms of cost-effectiveness and cases & death's reduction, it is found to be less effective than hand-hygiene & clean water distribution and treatment in most of the provinces in Zimbabwe (see **Tables 5**, **6** and **8** in **main text**). Cost per averted case of sanitation in most of the provinces is found to be higher than hand-hygiene & clean water distribution and treatment (see **Table 9** in **main text**). Thus, sanitation is a moderate cholera intervention which performs better than vaccination but less effective than hand-hygiene & clean water distribution and treatment.

Individual cost of hand-hygiene & clean water distribution, vaccination and sanitation are significantly reduced when applied with treatment. This reduction is almost one order in magnitude for most of the provinces for hand-hygiene & clean water distribution and sanitation. For vaccination, this reduction is one order in magnitude for Mashonaland Central, Mashonaland East, Midlands and Masvingo (see, **Table 8** in **main text**). Two order in magnitude for Harare, Matabeleland South and three order in magnitude for Mashonaland West and Manicaland (see, **Table 8** in **main text**). Significant amount of cost is reduced when vaccination is applied with hand-hygiene & clean water distribution and Sanitation; one order in magnitude for Mashonaland Central, Mashonaland East, Midlands and Masvingo; two order in magnitude for Harare, Mashonaland West and Matabeleland South and three order in magnitude for Manicaland (see, **Table 8** in **main text**).

References

1. Codeço CT (2001) Endemic and epidemic dynamics of cholera: the role of the aquatic reservoir. BMC Infect Dis: 1:1.
2. Islam MS, Draser BS, Sack RB (1994) Probable role of blue-green algae in maintaining endemicity and seasonality of cholera in Bangladesh: a hypothesis. J Diarrhoeal Dis Res 12(4): 245 - 56.
3. Alam M, Hasan NA, Sadique AK, Bhuiyan NA, Ahmed KU, et al (2006) Seasonal Cholera Caused by Vibrio cholerae Serogroups O1 and O139 in the Coastal Aquatic Environment of Bangladesh. Appl Environ Microbiol 72(6): 4096 - 4104.
4. Lipp EK, Huq A, Colwell RR (2002) Effects of Global Climate on Infectious Disease: the Cholera model. Clin Micro Rev 15(4): 757 - 770.

5. Sack RB, Sadique AK, Longini IM, Nizam A, Yunus M, et al (2003) A 4 year study of the epidemiology of Vibrio cholerae in four rural areas of Bangladesh. J Inf Dis 187(1): 96 - 101.
6. Colwell RR (1996) Global Climate and Infectious Disease: The Cholera Paradigm. Science 274(5295): 2025 – 2031.
7. Huq A, Colwell RR (1996) Environemntal factors associated with emergence of disease with special reference to cholera. East Mediter Heath J 2(1): 37 - 45.
8. Islam MS, Draser BS, Bradley DJ (1989) Attachment of toxigenic Vibrio cholerae O1 to various freashwater plants and survival with a filamentous green alga Rhizoclonium fontanum. J Trop Med Hyg 92(6): 396 - 401.
9. Glass R, Claeson M, Blake P, Waldman R, Pierce N (1991) Cholera in Africa: lessons on transmission and control for Latin America. Lancet 338(8770): 791 - 795.
10. Merrell DS, Butler SM, Qadri F, Dolganov NA, Alam A (2002) Host-induced epidemic spread of the cholera bacterium. Nature 417: 642 - 645.
11. Hartley DM, Morris JGJr, Smith DL (2006) Hyperinfectivity: A Critical Element in the Ability of V. cholerae to Cause Epidemics? PLoS Med 3(1): 63 - 69.
12. Lakshmikantham V, Leela S, Martynyuk AA (1989) Stability Analysis of Nonlinear Systems. Marcel Dekker Inc., New York and Basel.
13. Wang W, Zhao XQ (2008) Threshold Dynamics for Compartmental Epidemic Models in Periodic Environments. J Dyn Diff Equat 20(3): 699 - 717.
14. Mukandavire Z, Liao S, Wang J, Gaff H, Smith DL, et al (2011) Estimating the reproductive numbers for the 2008-2009 cholera outbreaks in Zimbabwe. PNAS 108(21): 8767 - 8772.
15. Aronsson G, Kellogg RB (1973) On a differential equation arising from compartmental analysis. Math Biosci 38: 113 - 122.
16. Hirsch MW (1985) Systems of differential equations that are competitive or cooperative II: convergence almost everywhere. SIAM J Math Anal 16: 423 - 439.
17. Zhang F, Zhao XQ, (2007) A periodic epidemic model in a patchy environment. J Math Anal Appl 325: 496 - 516.
18. Smith HL, Waltman P (1995) The Theory of the Chemostat: Dynamics of Microbial Competition. Cambridge Univ Press, Cambridge(England).
19. Thieme HR, (1992) Convergence results and a Poincaré-Bendison trichotomy for asymptotically autonomous differential equations. J Math Biol 30(7): 755 - 763.
20. Zhao XQ (2003) Dynamical Systems in Population Biology. Vol. 16; Springer-Verlag,, Canadian Mathematical Soceity.
21. Zhu Y, Wang K (2011) Existence and global attractivity of positive periodic solutions for a predator-prey model with modified Leslie-Gower Holling-type II schemes. J Math Anal Appl 384(2): 400 - 408.
22. Pontryagin LS, Boltyanskii VG, Gamkrelize RV, Mishchenko EF (1967) The Mathematical Theory of Optimal Process. Wiley, New York.
23. Fleming WH, Rishel RW (1975) Deterministic and Stochatic Optimal Control. Springer, Springer, Berlin.
24. Kirk DE (2004) Optimal Control Theory: An Introduction. Dover publications, Inc , Mineola, New York.
25. Chao DL, Halloran ME, Longini IM Jr (2011) Vaccination strategies for epidemic cholera in Haiti with implications for the developing world. Proc Natl Acad Sci U S A 108(17): 7081-85.
26. Andrews JR, Basu S, (2011) Transmission Dynamics and Control of Cholera in Haiti: An Epidemic Model. Lancet 377(9773): 1248 - 1255.
27. Naficy A, Rao MR, Paquet C, Antona D, Sorkin A, et al (1998) Treatment and vaccination strategies to control cholera in sub-Saharan refugee settings: a cost-effectiveness analysis. JAMA 279(7): 521-25.
